# The calcium-binding protein S100B reduces IL6 production in malignant melanoma via inhibition of RSK cellular signaling

**DOI:** 10.1371/journal.pone.0256238

**Published:** 2021-08-19

**Authors:** Milad J. Alasady, Alexander R. Terry, Adam D. Pierce, Michael C. Cavalier, Catherine S. Blaha, Kaylin A. Adipietro, Paul T. Wilder, David J. Weber, Nissim Hay

**Affiliations:** 1 Department of Biochemistry and Molecular Biology, University of Maryland School of Medicine, Baltimore, MD, United States of America; 2 Department of Biochemistry and Molecular Genetics, University of Illinois at Chicago, Chicago, IL USA; 3 Center for Biomolecular Therapeutics, University of Maryland School of Medicine, Baltimore, MD, United States of America; 4 University of Maryland Marlene and Stewart Greenebaum Comprehensive Cancer Center, Baltimore, MD, United States of America; University of Hawai’i at Manoa, UNITED STATES

## Abstract

S100B is frequently elevated in malignant melanoma. A regulatory mechanism was uncovered here in which elevated S100B lowers mRNA and secreted protein levels of interleukin-6 (IL6) and inhibits an autocrine loop whereby IL6 activates STAT3 signaling. Our results showed that S100B affects IL6 expression transcriptionally. S100B was shown to form a calcium-dependent protein complex with the p90 ribosomal S6 kinase (RSK), which in turn sequesters RSK into the cytoplasm. Consistently, S100B inhibition was found to restore phosphorylation of a nuclear located RSK substrate, CREB, which is a potent transcription factor for IL6 expression. Thus, elevated S100B reduces IL6-STAT3 signaling via RSK signaling pathway in malignant melanoma. Indeed, the elevated S100B levels in malignant melanoma cell lines correspond to low levels of IL6 and p-STAT3.

## Introduction

In vertebrates, normal S100B expression is cell-specific and found in chondrocytes, myoblasts, skeletal myofibrils, astrocytes, and melanocytes, but it is often highly overexpressed in cancers including most notably gliomas and malignant melanoma [1–3]. While several prognostic melanocytic biomarkers are available for melanoma such as the melanoma inhibitory activity (MIA) protein, lactate dehydrogenase (LDH), HMB 45, and Melan A/Mart 1, the S100B protein is regarded as being most useful since elevated serum levels of S100B correlate highly with poor patient response to therapies, increased cancer recurrence, and lower survival rates [4–7]. In addition to being a prognostic indicator for patient survival, S100B itself contributes to tumor progression when overexpressed by acting on several molecular targets including p53, Hdm2, HdmX, and the p90 ribosomal S6 kinase (RSK) [8–12].

Interleukin-6 (IL6) is a cytokine that exhibits both pro-inflammatory and anti-inflammatory activities [13–16]. IL6 was identified originally as a B-cell differentiation factor, but was later found to affect cell growth, activation, and differentiation of multiple immune cells such as B and T lymphocytes, monocytes, dendritic cells, and tumor-infiltrating lymphocytes [13, 16–21]. In general, IL6 signaling occurs via the binding of IL6 to either the cell membrane IL6 receptor (IL6R) or the soluble IL6 receptor (sIL6R) and then binds to the ubiquitously expressed gp130 receptor. It is the association of the (IL6/IL6R) dimer to gp130 that initiates auto-phosphorylation of an intracellular domain on gp130, the phosphorylation and activation of Janus Kinase (JAK1/2), and ultimately the activation of downstream transcription factors, such as STAT3, Akt1/2, and RAS/MAPK kinases [13, 22]. There are reports showing other IL6 binding partners (i.e. CD5) [23], but their mechanisms of action are not dilineated as extensively. Like many cytokines, IL6 can support or inhibit cellular proliferation depending on the cell type and the cellular environment. For example, IL6 signaling can block growth and proliferation of early stage melanoma [24–26] via promoting expression of genes essential to an immune response(s), which can target early-stage cancer cells for destruction [17–19, 21]. Data presented here show that elevated S100B inhibits IL6 production, by blocking CREB phosphorylation, which is known to activate IL6 expression and thus reduces STAT3 phosphorylation and transcripitional activity. With such data in mind, a model is discussed here in which elevated S100B may allow for melanoma cancer cells to escape immune detection by avoiding an early-stage IL6 dependent immune response in the tumor microenvironment.

## Materials and methods

### Cell culture

WM115 malignant melanoma cell lines were obtained from ATCC and subjected to Lentiviral shRNA Particle Infection targeting S100B as described elsewhere [8]. The cells were maintained in Minimal Essential Medium (MEM) supplemented with 10% heat-inactivated FBS, 1% Penicillin-Streptomycin at 100 units/ml, and 0.5 μg/mL Puromycin. WM793 and WM1158 melanoma cells were provided by Dr. David Kaetzel (University of Maryland School of Medicine) and maintained in MEM supplemented with 10% heat-inactivated FBS and 1% Penicillin-Streptomycin at 100 units/ml. SK-MEL-28 melanoma cells were maintained in DMEM (high glucose), 10% heat-inactivated FBS, and 1% Penicillin-Streptomycin at 100 units/ml. The coding sequence of human S100B was inserted into the Lenti-X pLVX TetOne puromycin vector (Clontech) and virus was packaged in HEK293T Lenti-X cells. After confirming viral titer, parental WM1158 were transduced with pLVX TetOne puro S100B or pLVX TetOne puro Luciferase as a control vector. Cells were grown under selection for several weeks in MEM supplemented with 10% Tetracycline-free FBS (Gemini) and 1% Penicillin-Streptomycin at 100 units/ml, in the presence of 2 μg/ml puromycin. Single colonies were picked, expanded and tested for the induction of S100B by exposing cells to Doxycycline at a final concentration of 2μg/ml for 24–72 hours and running a western blot to detect S100B levels.

### RT^2^PCR array

Total RNA was extracted from the cells using cold 1x PBS and subjected to RNA extraction using Plus RNeasy Mini kit (Qiagen). First strand cDNA synthesis was performed using RT^2^ First Strand Kit (Qiagen) with 0.5 μg total RNA as recommended by the manufacture. Real-time PCR was performed using RT^2^ SYBR Green qPCR Mastermix at volumes recommended by the manufacture. Data Analysis was performed using RT^2^ Profiler PCR Array Data Analysis provided by the manufacture at http://pcrdataanalysis.sabiosciences.com/pcr/arrayanalysis.php.

### In vitro STAT3 inhibitor (S31-201) treatments

Control WM115 and stable S100B knockdown cells were seeded in triplicate in 60 mm dishes at 7.0 x 10^5^ cells/dish in 1x MEM (Cellgro) supplemented with 10% FBS, 1% Penicillin-Streptomycin at 100 units/ml, 0.5μg/ml Puromycin and allowed to adhere overnight. The following day, the old media was removed and new media containing DMSO or 100 μM final concentration of S31-201 was added. The cells were incubated for 48 hours and then harvested at 48 hours post treatments using cold 1x PBS (Corning) or Trizol (Invitrogen) and subjected to western blotting and RNA extraction, respectively.

### siRNA transfection

Cells were seeded in 10 cm dishes at 5.0 x 10^5^ cells/dish and allowed to adhere overnight. The cells were then transfected with S100B siRNAs (Sigma Aldrich, siS100B-1: SASI_Hs01_00140287 and siS100B-2: SASI_Hs01_00140288), siCREB (Sigma Aldrich, SASI_Hs01_00116985) or control siRNA at 10 nM final concentration using siRNA Transfection Reagent following the protocol provided by the manufacture (Sigma Aldrich). The cells were incubated for 72 hours and then harvested with either cold 1x PBS or Trizol for Western blot analysis and RNA extraction, respectively.

### Dox-inducible system

WM1158 transfected with either empty vector or S100B-expressing vector were seeded in 60 mm dishes at 2.5 x 10^5^ cells/dish in 1x MEM (Cellgro) supplemented with 10% FBS, 1% Penicillin-Streptomycin at 100 units/ml, 2 μg/ml Puromycin and allowed to adhere overnight. The following day, the old media was removed and new media containing Doxycycline at 2 μg/mL final concentration was added. The cells were incubated for 72 hours. Subsequently, the old media was removed, replaced with fresh media, and incubated for 24 hours. Then, the cells were harvested with either 1x PBS (Corning) or Trizol (Invitrogen) and subjected to western blotting and RNA extraction, respectively.

### Conditioned medium assay

Stable S100B knockdown WM115 cells were growing for 5 days; the media was then harvested and spun down at 1000 rpm for 5 minutes. The Conditioned media (CM) was then added with either Normal Mouse IgG (Santa Cruz) or IL6 Inhibitory Antibody (R&D systems) at 0.15 μg/ml final concentration to Scrambled-WM115 cells or SK-MEL-28 cells. Cells were then harvested at 0, 2, 4, and 8 hours with cold 1x PBS, and subjected to Western blot analysis.

### Flow cytometry

Non-targeting scrambled and stable S100B knockdown WM115 cells were growing in MEM supplemented with 10% FBS, 1% Penicillin-Streptomycin at 100 units/ml, and 0.5 μg/mL Puromycin. Cells were detached using Trypsin, pelleted at 1000 rpm for 5 min, and media was removed. Cells were resuspended in 5 mL of Cell Staining Buffer (BioLegend Cat. No. 420201), centrifuged at 350 x g for 5 minutes, and supernatant was discarded. Cells were then resuspended in 200 μl of Cell Staining buffer. Cells were then incubated with 10 μl of either PE anti-human CD126 (IL-6R) antibody (Cat. No. 352803) or PE anti-human CD130 (gp130) Antibody (BioLegend Cat. No. 362003) and incubated on ice for 1 hour in the dark at 4 ˚C. Cells were then washed two times with Cell Staining Buffer and centrifuged at 350 x g for 5 mins. Then, cell pellets were resuspended in 150 μl of Cell Staining Buffer and 5 μl of 7-AAD Viability Staining Solution (BioLegend Cat. No. 420403) was added to exclude dead cells. Cells were incubated on ice for 15 mins in the dark at 4 ˚C. Flow Cytometry was performed using Beckman Coulter Epics Ellte ES (Beckman) and analyzed using FlowJo software.

### Immunoblot analysis

Immunoblotting analyses were performed using 25 μg total protein from the cell lysates. Protein concentrations was determined using the Bradford protein assay (Bio-Rad). Electrophoresis was performed using either an 8% or 12% SDS-PAGE gel and then subsequently transferred to PVDF membranes (Bio-Rad). The blots were and reacted with mouse anti-S100B (1:2000, BD Biosciences, Catalog No. 612376), rabbit anti-Phopho-STAT3 (1:1000, Cell signaling, Catalog No. 9145), rabbit anti-STAT3 (1:1000, Cell signaling, Catalog No. 12640), mouse anti-CREB (1:1000, Cell signaling, Catalog No. 9104), rabbit anti-Phospho-CREB (1:1000, Cell signaling, Catalog No. 9198), GAPDH (1:10,000, Cell signaling, Catalog No. 5174), β-actin (1:10,000, Sigma, Catalog No. A5541), rabbit anti-SP1 (1:1000, Cell signaling, Catalog No. 9389), rabbit anti-Phospho-MEK1/2 (1:1000, Cell signaling, Catalog No. 9121), mouse anti-vinculin (1:5000, Sigma, Catalog No. V9131); rabbit phospho-AKT (S473) (1:1000, Cell signaling Catalog No. 9271). Immobilon Western Chemiluminescent HRP Substrate (Thermo Fisher) was used at dilutions recommended by the manufacturer to detect proteins levels.

### Interleukin-6 ELISA

Media at the indicated time points and conditions were removed prior to harvesting cells, diluted in the assay buffer, and subjected to ELISA (Thermo Fisher, EH2IL6) as recommended by the manufacturer’s using recombinant human IL6 supplied for generating a standard curve.

### Real-time quantitative PCR

Total RNA was extracted at the indicated time points and conditions using Trizol (Invitrogen), and first strand cDNA synthesis was made using iScript cDNA synthesis kit (Bio-Rad). Quantitative real-time PCR was performed using iQ SYBR Green Supermix (Bio-Rad). The mRNA levels of target genes were measured relatively to the mRNA level of GAPDH. Primer sequences: SOCS3 5’-CATCTCTGTCGGAAGACCGTCA-3’ and 5’-GCATCGTACTGGTCCAGGAACT-3’; CEBPD 5’-TCCGGCAGTTCTTCAAGCAGCT-3’ and 5’-GAGGTATGGGTCGTTGCTGAGT-3’; IL6 5’-TGGATGCTTCCAATCTGGATTCAAT-3’ and 5’-AAATCTGTTCTGGAGGTACTCTAGGT-3’; CREB 5’-GAGCCGAGAACCAGCAGAGTG-3’ and 5’-AGTTACGGTGGGAGCAGATGATG-3’; GAPDH 5’-GACAGTCAGCCGCATCTTC-3’ and 5’-ACTCCGACCTTCACCTTCC-3’; S100B 5’-GAAGAAATCCGAACTGAAGAAGC-3’ and 5’- TCCTGGAAGTCACATTCGCCGT-3’; SOCS1 5’-TTCGCCCTTAGCGTGAAGATGG-3’ and 5’-TAGTGCTCCAGCAGCTCGAAGA-3’.

### Statistical analysis

Data are presented as mean ± SD as indicated in each figure. Differences in means were analyzed by using the Student’s *t*-test, one-way, and two-way ANOVA. All graphs and statistical analysis were generated using GraphPad Prism version 8 and a *p*-values of ≤ 0.05 were considered statistically significant.

## Results

### Elevated S100B in malignant melanoma decreases IL6 mRNA and secreted protein levels

**Fig 1:**
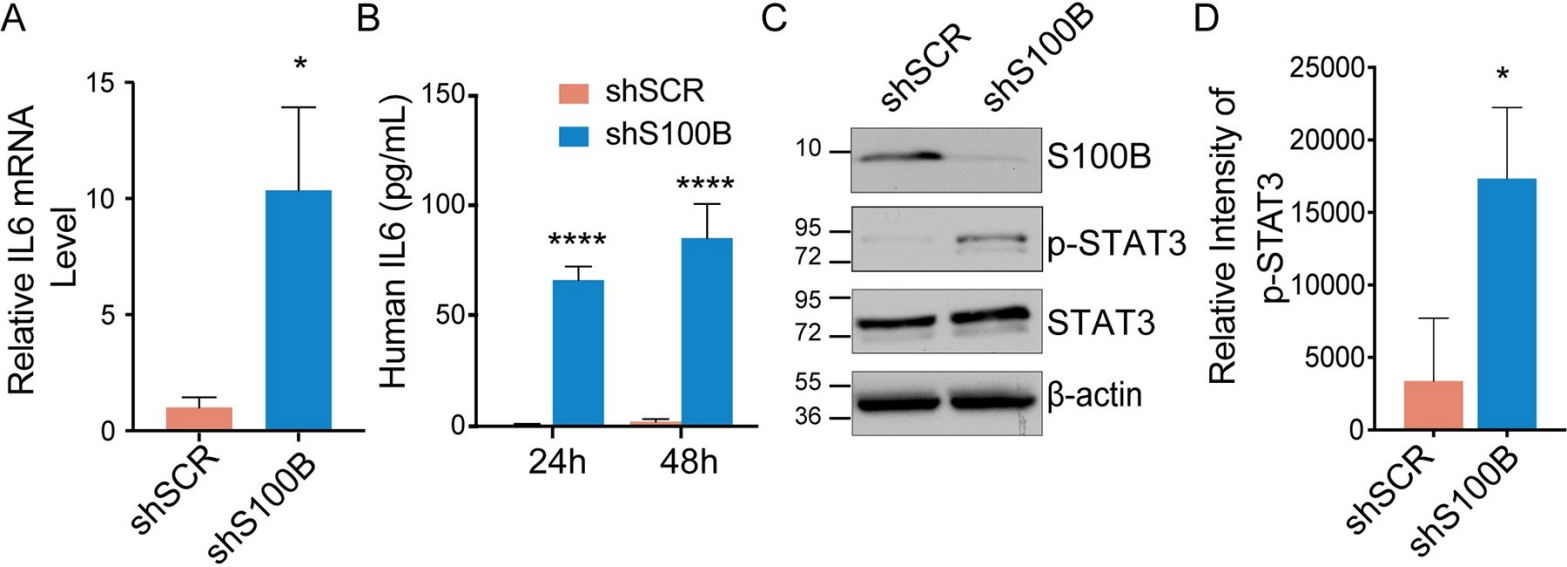
Knockdown of S100B in WM115 malignant melanoma cells increases IL6 expression and STAT3 phosphorylation. (A) qRT-PCR analysis was done to compare the IL6 mRNA levels in non-targeting scrambled and stable S100B knockdown WM115 cell lines, respectively and (B) an IL6 ELISA was used to detect secreted IL6 levels 24 and 48 hours after the introduction of new media. Both IL6 mRNA and secreted protein levels increase significantly upon knockdown of S100B (n = 4, mean ± SD; *, P < 0.05; ****, P < 0.0001). (C) Immunoblot analysis of shS100B WM115 cells shows little or no detectable S100B protein compared to shSCR WM115. In addition, levels of total STAT3 remain constant while there is a significant increase in p-STAT3 (Tyr705) in shS100B versus shSCR WM115 as shown by the average densitometry measurements of the p-STAT3 (D) (n = 3, mean ± SD; *, P < 0.05).

To further characterize the function of S100B in melanoma, quantitative real-time PCR arrays were used in S100B-targeting (shS100B) or non-targeting scrambled shRNA-expressing (shSCR) WM115 melanoma cells (shSCR and shS100B WM115 hereafter) [8], to identify genes that were either up- or down-regulated upon silencing of S100B expression. Using this approach, interleukin-6 (IL6) was discovered to be a top hit. An increase in the mRNA expression (Fig 1A; > 10-fold) and secreted protein levels (> 50-fold; Fig 1B) of IL6 were identified when S100B expression was silenced (Fig 1C, and S1A Fig). To further confirm these results and to mitigate any off- target effect from shRNA, transient silencing of S100B in WM115 cells via two unique functionally validated siRNA sequences also showed an increase of the mRNA expression of IL6 (S1B and S1C Fig). Furthermore, a small molecule S100B inhibitor, SC0025, which was discovered previously to bind S100B and mediate S100B-dependent killing in melanoma cells was used [27]. Treating WM115 cells with SC0025 also caused an increase in secreted IL6 protein and was consistent with inhibiting S100B, as anticipated (S1D Fig). These results further confirm a significant increase of IL6 expression following depletion or inhibition of S100B.

Whether S100B silencing affected downstream IL6 signaling was measured next by monitoring STAT3 phosphorylation, a known target in the IL6 signaling pathway [13, 22]. Consistent with restoring IL6 signaling, STAT3 phosphorylation at Tyr705 was increased with no observed effect in total STAT3 protein upon S100B knockdown stably (Fig 1C and 1D) or transiently (S1E and S1F Fig). Flow cytometry with WM115 cells was performed next to determine whether silencing of S100B expression induces IL6 receptor and/or gp130 receptor expression. Surprisingly, in these experiments, the expression of the IL6 and gp130 receptors were reduced in the stable S100B knockdown compared to shSCR WM115 cells (S2A and S2B Fig). This reduction in IL6/gp130 receptors upon S100B knockdown is consistent with a feedback mechanism in which elevated IL6 binds to the IL6R/gp130 receptor and induces its internalization, resulting in reduced IL6R/gp130 receptors on the membrane [28]. ELISA was used to measure levels of the soluble IL6 receptor (sIL6R) and the soluble gp130 receptor (sgp130) in the media in response to S100B knockdown. Although sIL6R was not detectable, sgp130 receptor was modestly increased in the stable S100B knockdown (S2C and S2D Fig). The modest increase in sgp130 is suggestive that S100B also inhibits IL6-trans signaling. Future experiments are required to determine whether S100B inhibits IL6-trans signaling via sgp130.

**Fig 2:**
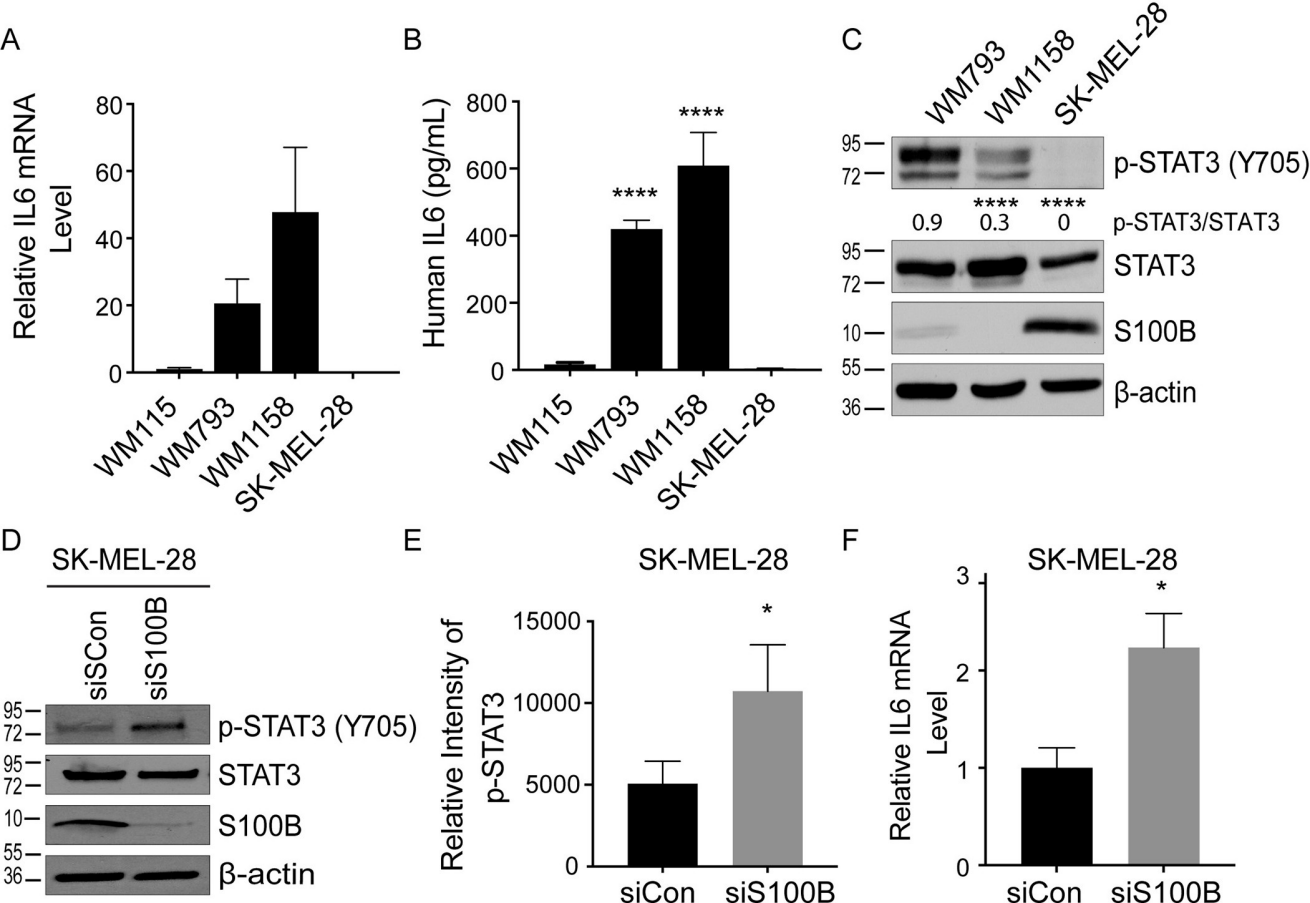
S100B levels changes IL6 and p-STAT3 in several melanoma cell lines. qRT-PCR used to measure the IL6 mRNA level (A) and an ELISA used to measure secreted IL6 protein level (B) show that S100B levels inversely correlate with IL6 mRNA and secreted protein levels in WM115, WM793, WM1158, and SK-MEL-28 cells (n = 3; mean ± SD; ****, P < 0.0001). (C) Immunoblot analysis of p-STAT3 (Tyr705), total STAT3, and S100B in WM793, WM1158, and SK-MEL-28 melanoma cells showing that varying levels of S100B inversely correlate with p-STAT3 (Tyr705) levels (n = 3; mean ± SD; ****, P < 0.0001). (D) Immunoblot analysis of p-STAT3, total STAT3, and S100B in SK-MEL-28 cells upon S100B knockdown using transient siRNA showed significant increases in p-STAT3 versus the control siRNA as represented graphically (E) (n = 3, mean ± SD; *, P < 0.05). (F) qRT-PCR shows IL6 mRNA in SK-MEL-28 cells transiently transfected with S100B targeting siRNA is significantly increased versus transfections with control siRNA (n = 3; mean ± SD; *, P < 0.05).

To study whether an inverse correlation between S100B and IL6 was a general phenomenon in melanoma, IL6 expression and STAT3 phosphorylation were measured in multiple melanoma cell lines with varied expression levels of endogenous S100B (i.e. WM793, WM1158, and SK-MEL-28). Consistent with the results of S100B knockdown in WM115 cells, WM793 and WM1158 cells that have little or no observable S100B protein had elevated IL6 expression (Fig 2A and 2B) and STAT3 phosphorylation at Tyr705 (Fig 2C). The SK-MEL-28 cells, which have high levels of S100B (Fig 2C), had comparatively lower levels of IL6 expression (Fig 2A and 2B) and STAT3 phosphorylation (Fig 2C). Controlled overexpression of S100B was achieved next in the WM1158 cells having low S100B via stable transfection with a doxycycline-inducible vector, and upon activation of S100B expression, there was a decrease in IL6 mRNA (S3A and S3B Fig), secreted IL6 protein level, and STAT3 phosphorylation (S3C, S3D and S3E Fig) again showing an inverse relationship between S100B and IL6 levels in malignant melanoma cell lines. Furthermore, S100B knockdown in SK-MEL-28 cells (Fig 2D) mirrored results from WM115 cells showing an increase in STAT3 phosphorylation (Fig 2D and 2E) and modest but statistically significant increase of IL6 mRNA (Fig 2F). Taken together, the data with WM115, WM793, WM1158, SK-MEL-28 melanoma cell lines support the conclusion that elevated S100B negatively regulates IL6 expression and its downstream signaling as measured via phosphorylation of STAT3. To further study the mechanism by which S100B inhibits IL6/STAT3 signaling, WM115 cells were used as a model system in the subsequent experiments.

### Elevated S100B decreases transcription of STAT3 regulated genes

**Fig 3:**
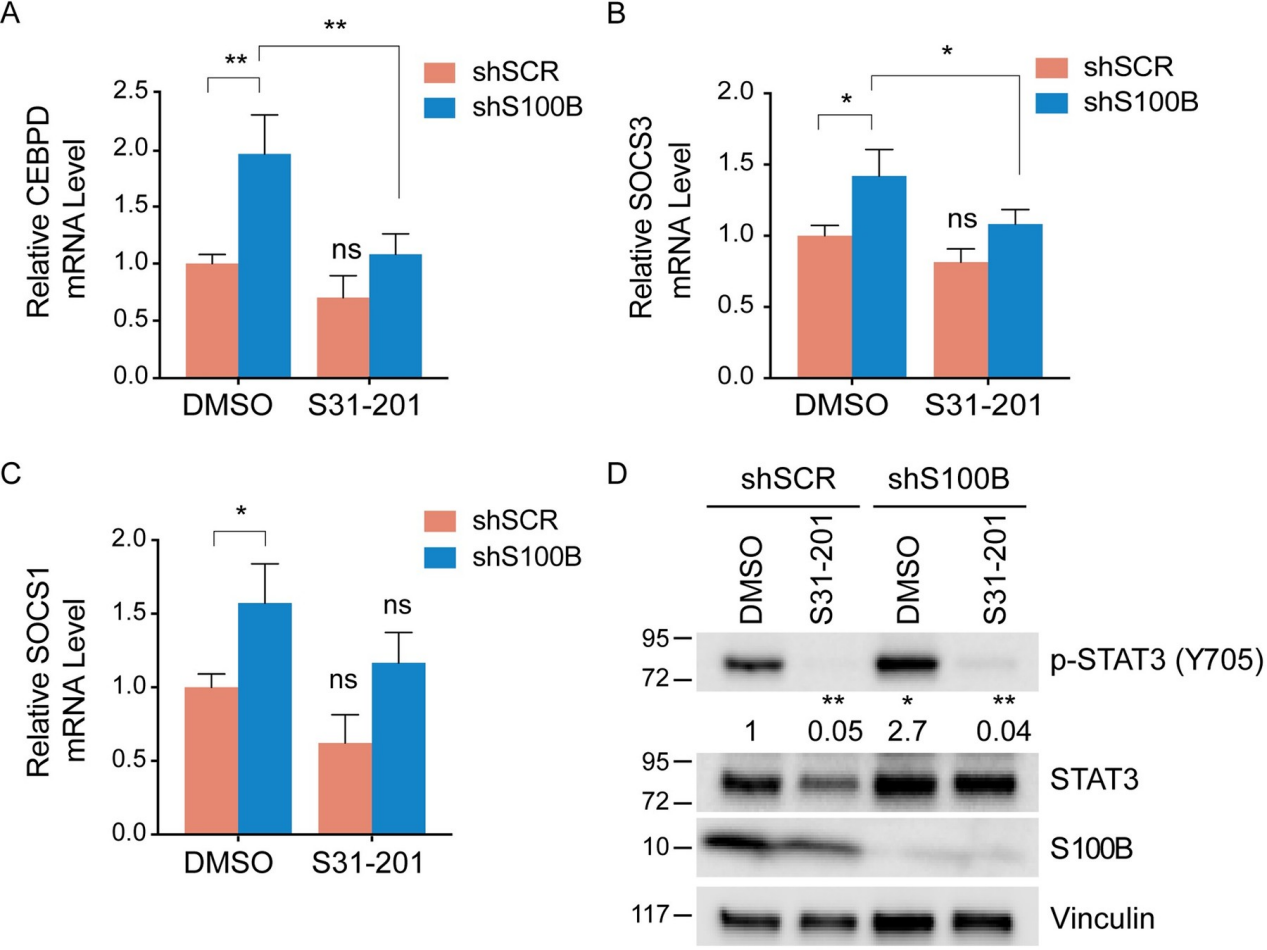
S100B inhibits transcription activity of p-STAT3. qRT-PCR was performed on STAT3 target genes CEBPD (A), SOCS3 (B), and SOCS1 (C) in shSCR and shS100B WM115 cells in the absence or presence of a STAT3 inhibitor S31-201 for 48 hours of treatment (100 μM). The knockdown of S100B results in increased transcription of all three p-STAT3 regulated genes. The introduction of the STAT3 inhibitor negated that effect for CEBPD and SOCS3 but was not statistically significant for SOCS1 (P = 0.07). (n = 3; mean + SD; *, P < 0.05; **, P < 0.005). (D) Immunoblot analysis of p-STAT3 (Tyr705) and S100B in shSCR and shS100B WM115 cells following 48 hours of S31-201 treatments (100 μM) confirmed the loss of p-STAT3 (Tyr705). (n = 3; mean + SD; *, P = 0.05; **, P < 0.005). Relative p-STAT3 protein levels were quantified using Image J software.

The transcription factor STAT3 is activated by phosphorylation at Tyr705, which induces its dimerization, nuclear translocation, and DNA binding [22]. To determine if transcriptional activity of STAT3 is inhibited by elevated S100B, the expression of three STAT3-target genes (i.e. CEBPD, SOCS3, and SOCS1) was measured using quantitative RT-PCR in shSCR and shS100B- WM115 cells. Consistent with an increase in STAT3 activity, knockdown of S100B increased the mRNA levels of all three STAT3-target genes compared to the scrambled control (Fig 3A–3C). When cells were treated with S31-201, a small molecule inhibitor of STAT3, CEBPD and SOCS3 mRNAs levels were attenuated in shS100B WM115 cells, further demonstrating that S100B knockdown induced expression of CEBPD and SOCS3 in a STAT3-dependent manner (Fig 3A and 3B). The mRNA levels of SOCS1 was also attenuated to some degree when treated with S31-201, but it was not statistically significant (Fig 3C). As expected, STAT3 inhibition with S31-201 reduced p-STAT3 in both control shSCR and shS100B WM115 cells without affecting total STAT3 (Fig 3D). However, the effect of STAT3-inhibition on STAT3 target-genes was greater in shS100B WM115 cells (Fig 3A, 3B and 3C). S31-201 had a minimal effect in the control shSCR WM115 cells, where both level and transcriptional activity of STAT3 phosphorylation were reduced. Together, these studies show that elevated S100B suppresses IL6 and STAT3 transcriptional activity in malignant melanoma.

### STAT3 activation requires IL6 autocrine activity

**Fig 4:**
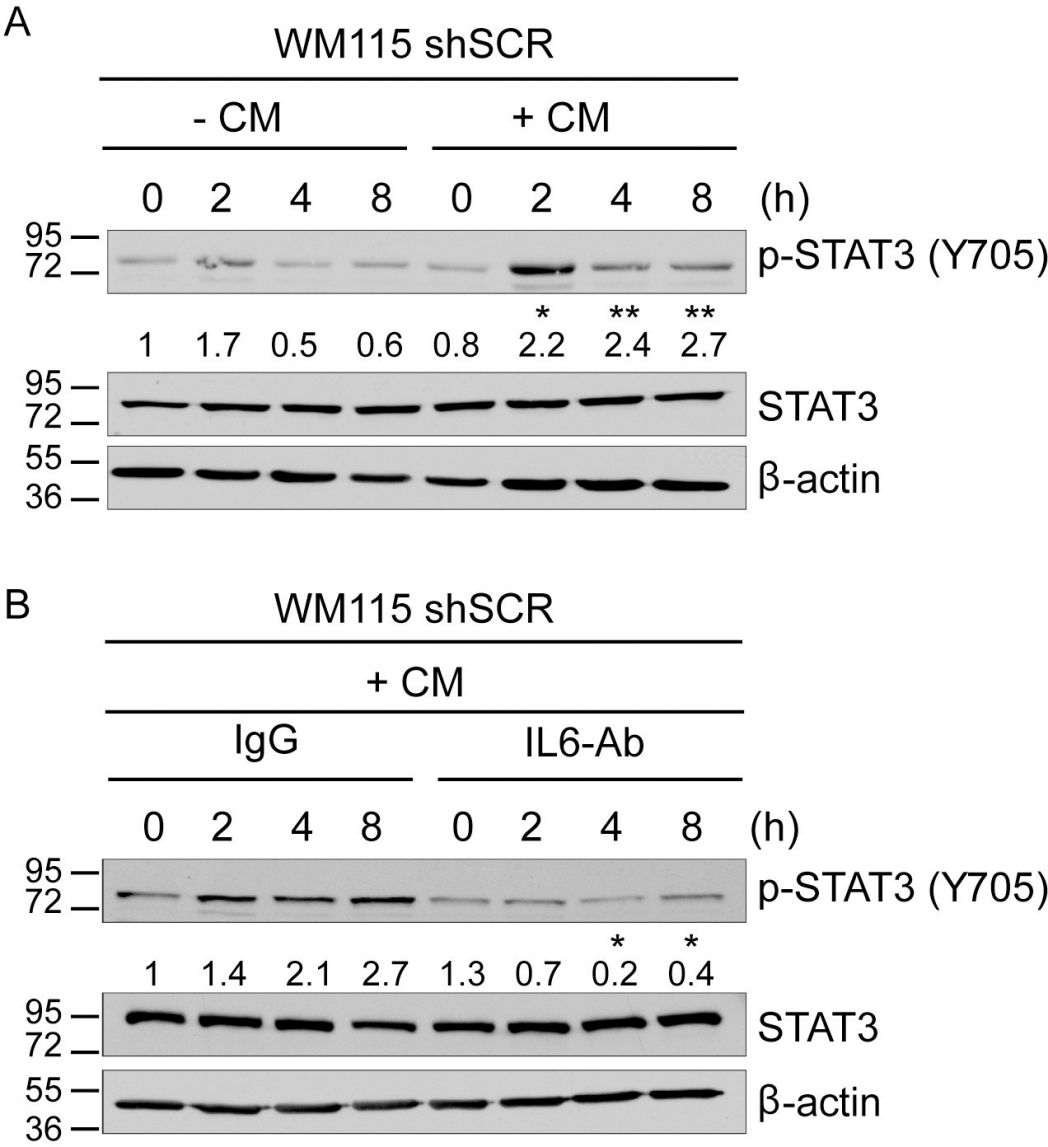
Restoration of p-STAT3 by the knockdown of S100B occurs via IL6 autocrine activity. A schematic design of this experiment is also available (S4 Fig). (A) Immunoblot analysis was done on shSCR WM115 cells after 0, 2, 4, and 8 hours of treatment with either shS100B cell conditioned media (shS100B CM) or control media. The conditioned media increases p-STAT3 as compared to the control (n = 3; mean ± SD; *, P < 0.05, **, P < 0.005). (B) shSCR WM115 cells were cultured with shS100B CM in the absence/presence of an IL6 inhibitory antibody (IL6 Ab; 0.15 μg/mL). Immunoblot analysis of p-STAT3 (Tyr705) and total STAT3 after 0, 2, 4, and 8 hours of treatment shows the IL6 Ab prevents increased p-STAT3 production compared to an equivalent concentration of a non-specific control IgG (n = 3; mean ± SD; *, P < 0.05). Relative p-STAT3 protein levels were quantified using Image J software.

To determine if S100B affects IL6/STAT3 signaling through an autocrine mechanism via secreted IL6, shSCR and shS100B WM115 cells were treated with an IL6 inhibitory antibody (IL6 Ab; Schematic in S4 Fig). The introduction of an IL6 antibody (IL6-Ab) to the growth media of the shS100B WM115 cells reduced p-STAT3 to levels similar to what was observed in the shSCR WM115 cells (S5A Fig). Conversely, conditioned media harvested from shS100B cells induced STAT3 phosphorylation (Tyr705) in shSCR WM115 cells, with a peak effect at 2h (Fig 4A). However, when an IL6-inhibitory antibody was added to this conditioned media, p-STAT3 induction was blocked (Fig 4B), demonstrating that secreted-IL6 is directly responsible for increased STAT3-activity in shS100B WM115 cells. Similar results were observed in SK-MEL-28 cells treated with shS100B WM115 conditioned media in the presence and absence of IL6 inhibitory antibody, indicating that this paracrine effect is not unique to WM115 cells (S5B Fig). These data indicate that the STAT3 activation in the shS100B WM115 cells was indeed via an IL6-dependent autocrine mechanism. Collectively, these data continue to support a mechanism in which elevated S100B found in malignant melanoma negatively regulates the IL6/STAT3 pathway and that functional IL6 can be restored when S100B expression is inhibited.

### S100B levels in malignant melanoma have little, if any, effect on IL6 mRNA stability

It is established that one mechanism for regulating cytokine levels, including IL6, can be via regulation of its mRNA stability [29]. Therefore, IL6 mRNA stability was measured via an Actinomycin D time-course assay to determine whether S100B knockdown increases the IL6 mRNA half-life. These experiments revealed that S100B knockdown had little if any measurable effect on the half-life of the IL6 mRNA in shS100B WM115 cells (half-life: 1.7 h ± 0.4 h) when compared to the shSCR WM115 cells (half-life: 1.2 h ± 0.3 h; S6 Fig).

### S100B reduces IL6 levels via an RSK-dependent mechanism

**Fig 5:**
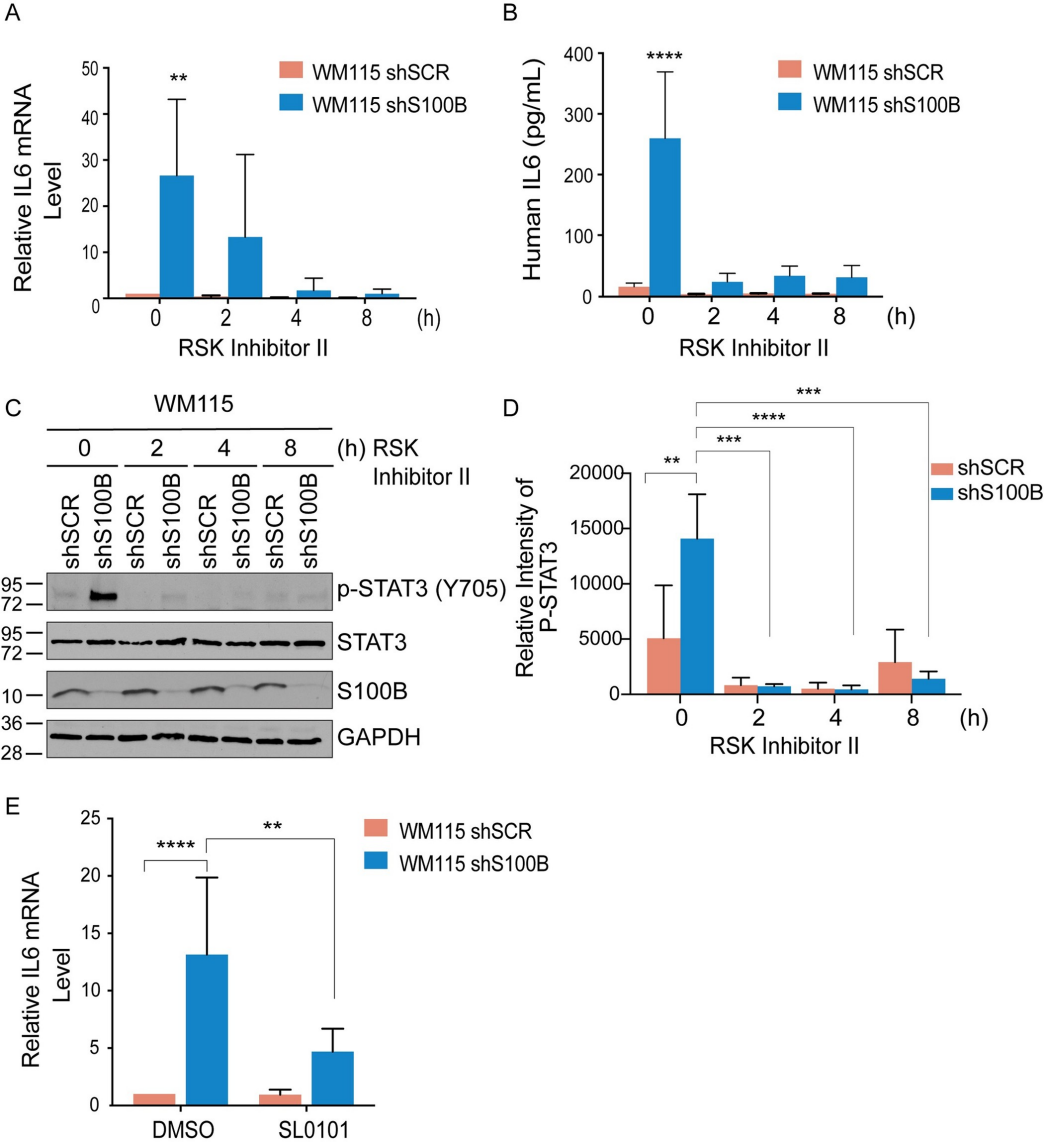
S100B regulation of IL6/p-STAT3 requires RSK. (A) qRT-PCR analysis of the IL6 mRNA and (B) ELISA of secreted IL6 protein in the media were performed on shSCR and shS100B WM115 cells after a 0, 2, 4, and 8 hours of treatment with RSK Inhibitor II, BI-D1870 (10 μM). (n = 3, mean ± SD; **, P < 0.005; ****, P < 0.0001). (C) Immunoblot analysis of the p-STAT3 (Tyr705), total STAT3, and S100B in shSCR and shS100B WM115 cells following 0, 2, 4, and 8 hours of RSK Inhibitor II treatments (10 μM) confirms that the RSK inhibitor prevents p-STAT3 (Tyr705). (D) The average values of relative p-STAT3 of three independent experiments were plotted using Image J (n = 3; mean ± SD; *, P < 0.05; **, P < 0.005; ****, P < 0.0001). (E) The inhibition of RSK with a second inhibitor (SL0101) also prevented the knockdown of S100B from restoring IL6 mRNA after 4 hours of inhibition with 10 μM of SL0101 as was found with the other RSK inhibitor, BI-D1870. (n = 6; mean ± SD; **, P < 0.005; ****, P < 0.0001).

It is important to define a mechanism for how elevated S100B in malignant melanoma suppresses the IL6/STAT3 pathway. While IL6 is activated by numerous transcription factors and cis- or trans-acting elements that are poised to respond to cellular cues, one such transcription factor, CREB, was of interest here because (i) it is a downstream target of RSK [30, 31] and (ii) RSK is regulated via a direct interaction with S100B [8]. In response to external stimuli, RSK activation and nuclear localization occur via sequential phosphorylation at Ser and Thr amino acids sites [32]. In previous work, Hartman et al. 2014 showed in malignant melanoma that S100B binds RSK in a calcium-dependent manner, sterically blocks ERK-dependent phosphorylation of RSK at Thr573, and sequesters RSK to the cytoplasm, and that RSK is re-localized to the nucleus following S100B depletion [8, 33]. Thus, Hartman et al. has previously showed that S100B directly binds and blocks RSK nuclear localization independent of MEK activation [8]. Therefore, to address whether or not RSK was also involved in the S100B-dependent repression of IL6 expression, a small molecule RSK inhibitor (BI-D1870) was administered to shSCR and shS100B WM115 cells (Fig 5). Interestingly, BI-D1870 treatment reduced IL6 mRNA and secreted protein levels quickly (< 2 hr) in shS100B WM115 cells when compared to shSCR WM115 control cells (Fig 5A and 5B). Phosphorylation of STAT3, which is downstream of IL6 production, was also decreased upon RSK-inhibition with no effect on total STAT3 levels (Fig 5C and 5D). To confirm the reduced level of IL6 mRNA from BI-D1870, a second inhibitor selective for RSK, SL0101, was administered to WM115 melanoma cells. Similarly, SL0101 treatment also decreased IL6 mRNA levels in the shS100B WM115 cells, and it did so much more robustly than shSCR WM115 cells (Fig 5E).

**Fig 6:**
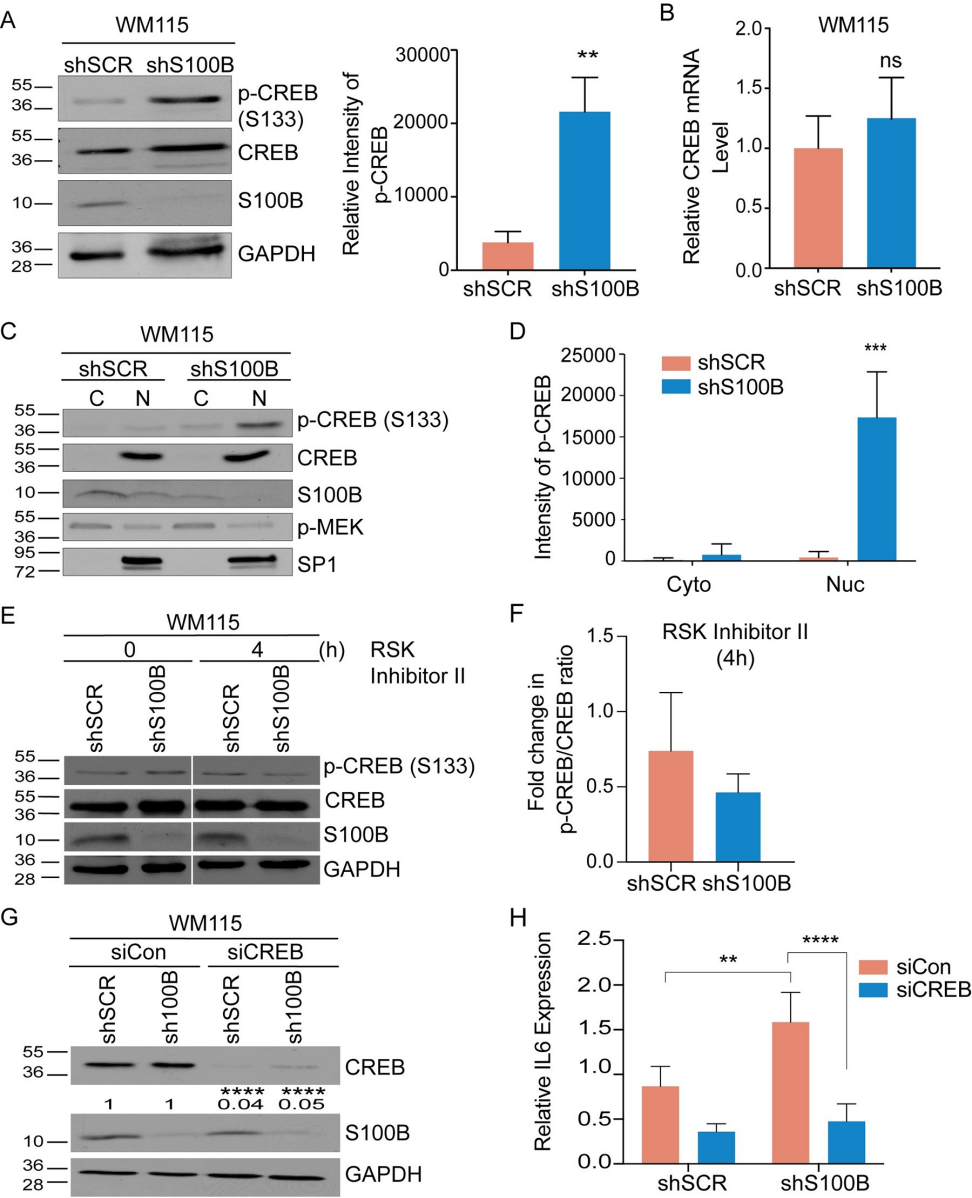
S100B suppresses phosphorylation and nuclear localization of CREB. (A) Immunoblot analysis of p-CREB (Ser133), in the shSCR and shS100B WM115 cells shows an increase in p-CREB (Ser133) upon S100B depletion without a significant increase in total CREB protein (n = 3; mean ± SD; **, P < 0.005) or (B) mRNA levels in the shSCR and shS100B WM115 cells (n = 4, mean ± SD; P = NS; non-significant). (C) Immunoblot analysis of nuclear (N) and cytoplasmic (C) fractions of shSCR and shS100B WM115 cells shows that nuclear located p-CREB (Ser133) is restored upon S100B depletion, with little if any CREB observed in the cytoplasm. Also shown are S100B levels, MEK (cytoplasmic marker), and SP1 (nuclear marker). (D) The average values of p-CREB intensity were plotted using image J (n = 3; mean ± SD; ***, P < 0.005). (E) Immunoblot analysis of the p-CREB (S133), total CREB, and S100B in shSCR and shS100B WM115 cells following 0 and 4 hours of RSK Inhibitor, BI-D1870 treatment (10 μM). (F) The fold change of p-CREB/total CREB ratio following 4 hours of RSK inhibitor, BI-D1870, were plotted using image J. (G) Transient knockdown of CREB in the shSCR and shS100B WM115 cells with either control or two different CREB siRNAs (n = 3; mean ± SD; ****, P < 0.0001. (H) qRT-PCR of IL6 mRNA in shSCR and shS100B WM115 cells transfected with either control or CREB siRNA shows loss of IL6 transcription with CREB knocked down (n = 3; mean ± SD; **, P < 0.005; ****, P < 0.0001).

**Fig 7:**
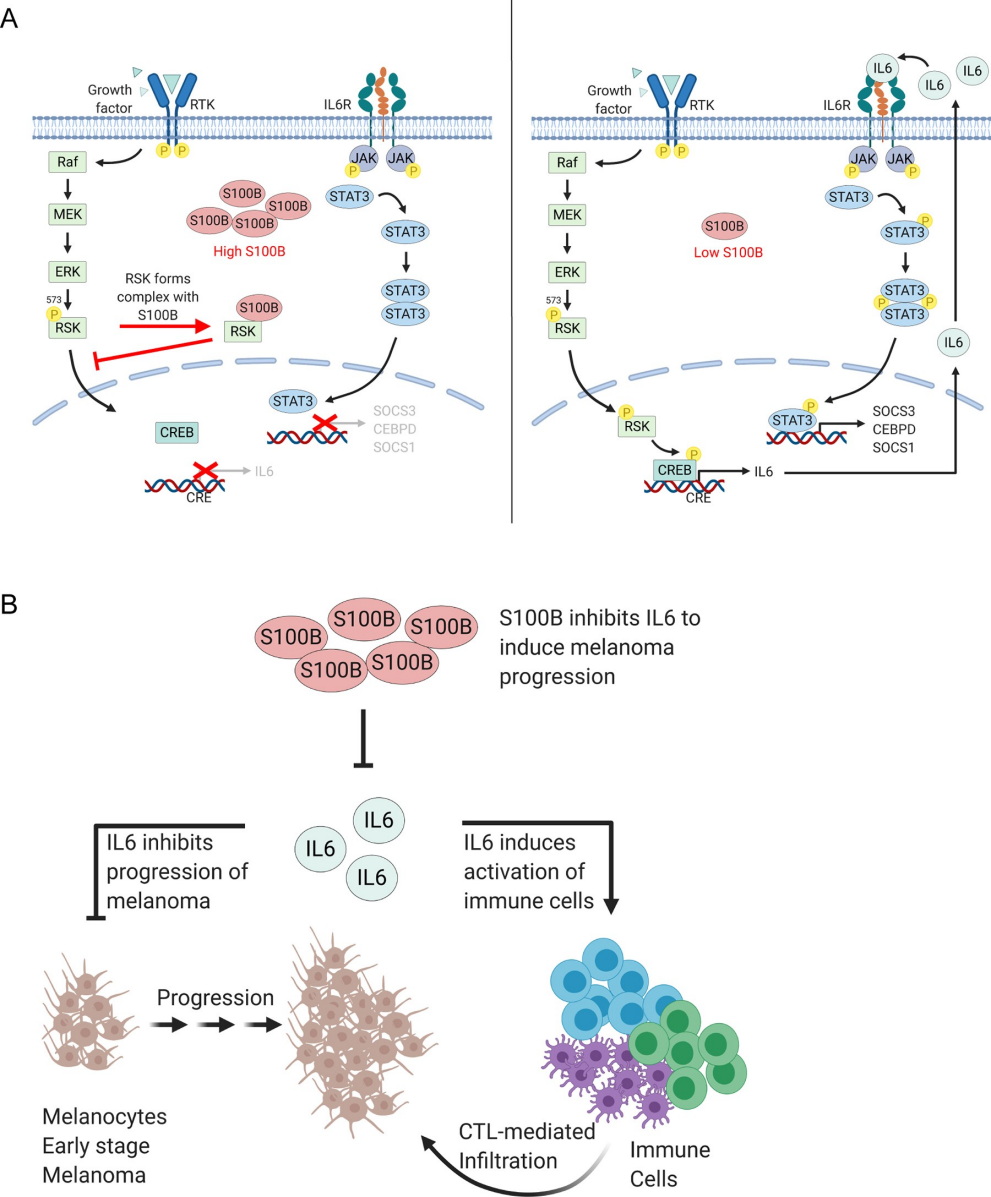
Model for S100B-dependent inhibition of IL6/STAT3 signaling and its possible role in the pathology of malignant melanoma. (A) Elevated levels of S100B found in most melanoma binds RSK in a calcium-dependent manner and sterically prevents its ERK-dependent phosphorylation at T573, which is needed for RSK nuclear localization (8). As a result, it is proposed here that elevated S100B inhibits expression and secretion of IL6 and phosphorylation and transcriptional activity of STAT3 (Figs 1–4). S100B-dependent inhibition of IL6 and STAT3 phosphorylation is via RSK (Fig 5). S100B also inhibits phosphorylation of CREB in the nucleus. Given that S100B forms a complex with RSK and prevents RSK nuclear localization, it is proposed that S100B inhibits phosphorylation of CREB in the nucleus via RSK (Fig 6). Thus, CREB-dependent expression of IL6 is inhibited, as is IL6/STAT3 signaling. (B) During early stages of melanoma progression, it is hypothesized that IL6 induces anti-proliferative mechanism to inhibit the progression of melanoma. Thus, when S100B levels elevate to a critical threshold in a pre-metastatic state, it blocks IL6 expression and initiates the transition of melanoma from pre-malignant to a malignant form.

Since CREB is a known substrate of RSK [34] and because it is a transcriptional activator for IL6 expression [35], its role in the S100B-dependent regulation of IL6 was examined next. Interestingly, silencing S100B expression in WM115 cells caused a large increase in CREB phosphorylation at Ser133 without significantly affecting its mRNA or total CREB protein levels (Fig 6A and 6B). As a control, p-CREB localization was tested as a function of S100B. While CREB was localized in the nucleus for both S100B proficient and deficient cells, phosphorylated CREB (at Ser133) was only elevated in the nuclear fraction of the shS100B WM115 cells (Fig 6C and 6D). Furthermore, RSK inhibitor (BI-D1870) treatment reduced phosphorylated CREB in the shSCR and shS100B WM115 cells with no effect on total CREB levels (Fig 6E and 6F). The role of CREB on IL6 expression in the stable S100B knockdown WM115 cells was confirmed next by blocking its expression via siRNA (i.e. a double knockdown; S100B & CREB knockdown). As predicted, IL6 mRNA was significantly suppressed in the stable S100B knockdown in WM115 cells in response to CREB knockdown as compared to cells transfected with scrambled siRNA (Fig 6G and 6H). These data are fully consistent with a mechanism in which high S100B levels found in malignant melanoma bind RSK and block its entry into the nucleus [8], which prevents the phosphorylation of nuclear-localized CREB and reduces expression of IL6 and IL6/STAT3 signaling (Fig 7A).

## Discussion

Promising treatment options for malignant melanoma (MM) patients involve immune checkpoint inhibitors, which are extremely effective for 20–50% of patients with type 1-immunity profiles (>10 year survival; i.e. for ipilumimab) [36]. Other effective therapies include chemotherapy, BRAF/MEK inhibitors, cytokines (i.e. interferon-α, interleukin-2), MM vaccines, and combinatorial approaches. Despite recent progress, the long-term prognosis (>5 yrs) remains poor for most MM patients (>50%), so efforts to delineate mechanisms underlying MM and to develop novel therapeutic(s) are still greatly needed [37]. The tumor marker S100B represents a novel MM target since it is elevated in >90% of patients, it correlates directly with poor survival [38, 39], contributes to the degradation of wild type p53 tumor suppressor [9–12, 40–56] and may contribute to immune evasion, particularly as recognized in melanoma vaccine clinical trials [2, 39, 57, 58]. However, the mechanism of action of S100B in cancer is still poorly understood. For these reasons, consequences of elevated S100B in malignant melanoma were studied further here.

Specifically, it was found that elevated S100B in malignant melanoma cells blocks CREB phosphorylation and inhibits IL6/STAT3-signaling (Fig 7A). Thus, nuclear p-CREB (Ser133), IL6 expression, and IL6/STAT3 signaling were all restored upon S100B knockdown in WM115 melanoma cells (Figs 6 and 7A). The data presented here are also fully consistent with a mechanism reported earlier [8] in which high S100B levels found in malignant melanoma bind to RSK, block its phosphorylation at T573, and sequester this enzyme to the cytoplasm [8]. As a result, it is proposed that phosphorylation of CREB in the nucleus is blocked by S100B-RSK complex (Fig 6), and CREB-dependent transcription of IL6 is inhibited, leading to the loss of IL6/STAT3 signaling in melanoma (Fig 7A).

IL6 is involved in the progression and development of melanoma, but the specific function(s) remain unclear. IL6 is deregulated in many cancers, with increased serum levels indicating a poor prognosis [24]. In canonical cis-signaling, IL6 appears pro-tumorigenic, with downstream activation of STAT3 stimulating proliferation, and inhibition of apoptosis. However, in certain cancer cells types, IL6 signaling was shown to be anti-proliferative [25, 26, 59, 60]. For example, in melanocytes and early stages of melanoma, IL6 was shown to inhibit cell growth [25, 26, 61–64]. Therefore, it is plausible that inhibition of IL6/ STAT3 pathway by elevated S100B in melanoma cells could be beneficial to early stage tumors as a way to prevent anti-proliferative effects of IL6 and to avoid immune-related destruction.

It was reported that melanoma cells undergo TNF-α-dependent apoptosis after treatments with IL6 and the soluble form of the IL6 receptor, sIL6R [65]. Using the mouse melanoma cell line B16F10.9, which are usually resistant to TNF-α-mediated cell death, apoptosis was enhanced in the presence of IL6/sIL6R. Additionally, using melanoma cell line A375, growth was inhibited by IL6 in a STAT3-specific manner [66]. This growth inhibition could be enhanced with additional treatment with sIL6R, once more indicating a role of IL6 in mediating growth inhibition at early stages of malignant melanoma. The results of this study with S100B silencing are consistent with these studies since slight increases of sgp130 are also observed here, which provide a possible role for S100B in negatively regulating the IL6-trans signaling (S2C Fig). Nevertheless, our current data suggest that elevated S100B may drive a mechanism, at least in part, to overcome the anti-proliferative effect of IL6/STAT3 signaling in pre-malignant melanoma cells and to induce the transition from the pre-malignant to the malignant stage of melanoma and support the observation that elevated S100B correlates with poor patient survival (Fig 7B).

Another benefit to tumor cells from S100B-dependent inhibition of IL6 secretion could be to prevent IL6 activation of B cells, T lymphocytes, and dendritic cells as a means to avoid an anti-tumor immune response at an early stage or in regions with little or no signs of a stress-response. A series of reports have shown that IL6 trans-signaling plays a major role in the immune response of the tumor cells by activating T-lymphocytes, promoting T-cells trafficking to the lymph nodes, transmigration of T lymphocytes into tumor tissues, and B-cells activation [13, 16, 18–21, 67]. In addition, the activation of a subset of cytotoxic T cells, tumor-infiltrating lymphocytes, against melanoma tumors was enhanced by the use of IL6 [68, 69]. Thus, the S100B-dependent inhibition of IL6 expression and secretion into a stress-free microenvironment could facilitate melanoma cells to escape the immune surveillance during melanoma progression (Fig 7B).

S100B levels are highly elevated in melanoma. However, the different roles of S100B in supporting growth and survival of malignant melanoma are not fully understood. The study reported here supports a role for elevated S100B levels in suppressing IL6/STAT3 signaling providing additional evidence of S100B in supporting cancer.

## Supporting information

S1 Fig S100B inhibits IL6 expression and STAT3 phosphorylation in WM115 transfected with S100B siRNA. (A) qRT-PCR of the relative S100B mRNA level in shSCR and shS100B WM115 cells (n = 3; mean ± SD; ***, P < 0.005). (B) Immunoblot analysis of S100B in the parental WM115 cells transfected with either control or S100B siRNAs (n = 3; mean ± SD; ***, P < 0.005). Relative S100B protein levels were quantified using Image J. (C) qRT-PCR of the relative IL6 mRNA level in the parental WM115 cells transfected with either control or S100B siRNA #1 and siRNA #2 (n = 3; mean ± SD; *, P < 0.05). (D) IL6 ELISA was used to detect secreted IL6 levels following treatment with either DMSO or S100B inhibitor (SC0025) at 15 μM for 48 hours (n = 3; mean ± SD; *, P < 0.05). (E) Immunoblot analysis of p-STAT3 (Tyr705), total STAT3, p-Akt (Ser473), and S100B in the WM115 transfected with either control or S100B siRNAs. (F) The average values of three independent experiments were plotted using Image J (n = 3; mean ± SD; *, P < 0.05).
(TIF)

S2 Fig Expression of the IL6 and gp130 receptors in WM115 melanoma cells. Expression of IL6R (A) and gp130 (B) receptors as determined by flow cytometry in non-targeting scrambled WM115 cells and stable S100B knockdown WM115 cells. Quantitative representation of the flow cytometry analysis (A and B) was done using Prism (n = 3; mean ± SD; ** P <0.005). ELISA was done to determine the secreted sIL6R (C) and sgp130 (D) in the media of the non-targeting scrambled and stable S100B knockdown WM115 cells; n = 2; ± SD; *, P < 0.05).
(TIF)

S3 Fig S100B overexpression reduces IL6 and p-STAT3 in WM1158 cell line. The overexpression of S100B in the WM1158 cells stably expressing a tetracycline-inducible S100B vector (B) shows a significant decrease in relative IL6 mRNA levels via qRT-PCR while an empty vector control (A) had no significant effect (n = 3; mean ± SD.; *, P < 0.05; NS, non-significant). The cells were induced for 72 hours with 2 μg/mL Doxycycline (Dox). (C) ELISA was used to measure secreted IL6 in the WM1158 cells stably expressing tetracycline-inducible S100B vector (WM1158-S100B) showing decreased of secreted IL6 protein level in response to S100B expression (n = 3; mean ± SD.; ***, P < 0.0005). (D) Immunoblot analysis showed an increased S100B upon Dox treatment and decrease in p-STAT3 (Tyr705) with no effect on total STAT3 as shown by the average densitometry measurements of the p-STAT/total STAT3 ratio (E) relative p-STAT3 protein levels were quantified using Image J software (n = 3; mean ± SD; *, P < 0.05).
(TIF)

S4 Fig Schematics for experimental design used for data presented in Fig 4. (A) Diagram representing the experimental design for WM115 shSCR and shS100B WM115 cells treated with IgG or IL6-Ab in Fig 4A. (B) Diagram representing the experimental design for the conditioned media (CM) experiment in shSCR WM115 cells in Fig 4B. (C) Diagram representing the experimental design for the conditioned media experiment (CM) in the presence of IgG or IL6-Ab in the shSCR WM115 and SK-MEL-28 cells in Fig 4C and 4D.
(TIF)

S5 Fig p-STAT3 is restored following S100B depletion via IL6 autocrine activity. (A) The IL6 inhibitory antibody (0.15 μg/mL) prevented the autocrine activation of p-STAT3 upon addition to shS100B WM115 cells growth media compared to shSCR WM115. After 8 hours, the IL6 Ab treated shS100B cells did not have the increased p-STAT3 seen in the control IgG treated shS100B cells. (B) SK-MEL-28 cells were cultured with the shS100B WM115 CM in the absence/presence of an IL6 Ab (0.15 μg/mL). Immunoblot analysis of p-STAT3 (Tyr705) and total STAT3 after 0, 2, 4, and 8 hours of treatment shows the IL6 Ab prevents increased p-STAT3 production. Relative p-STAT3 protein levels were quantified using Image J software. Blot is a representative of two independent replicates.
(TIF)

S6 Fig S100B does not change the stability of IL6 mRNA. The mRNA half-life of IL6 mRNA was measured in the non-targeting scrambled (shSCR) and S100B stable knockdown (shS100B) cell lines by Actinomycin D assay. The percentage of IL6 mRNA remaining was plotted as a function of time following transcription inhibition by Act D. Plot is an average of two independent biological replicates. The data were fit to one phase exponential decay using Prism 8.0 to calculate the half-life of IL6 mRNA. shSCR half-life: 1.2 h ± 0.4 h; shS100B half-life: 1.7 h ± 0.3 h; P = 0.1908. The standard errors were calculated from the 95% confidence intervals. The goodness of fit with R^2^ for the rates was 0.84 (shSCR) and 0.88 (shS100B).
(TIF)

S1 Raw Images (PDF)
