# Supplementary figures and images for: The calcium-binding protein S100B reduces IL6 production in malignant melanoma via inhibition of RSK cellular signaling
Source: PLoS One. 2021 Aug 19;16(8):e0256238. doi: 10.1371/journal.pone.0256238 (PMC8376063; doi:10.1371/journal.pone.0256238)

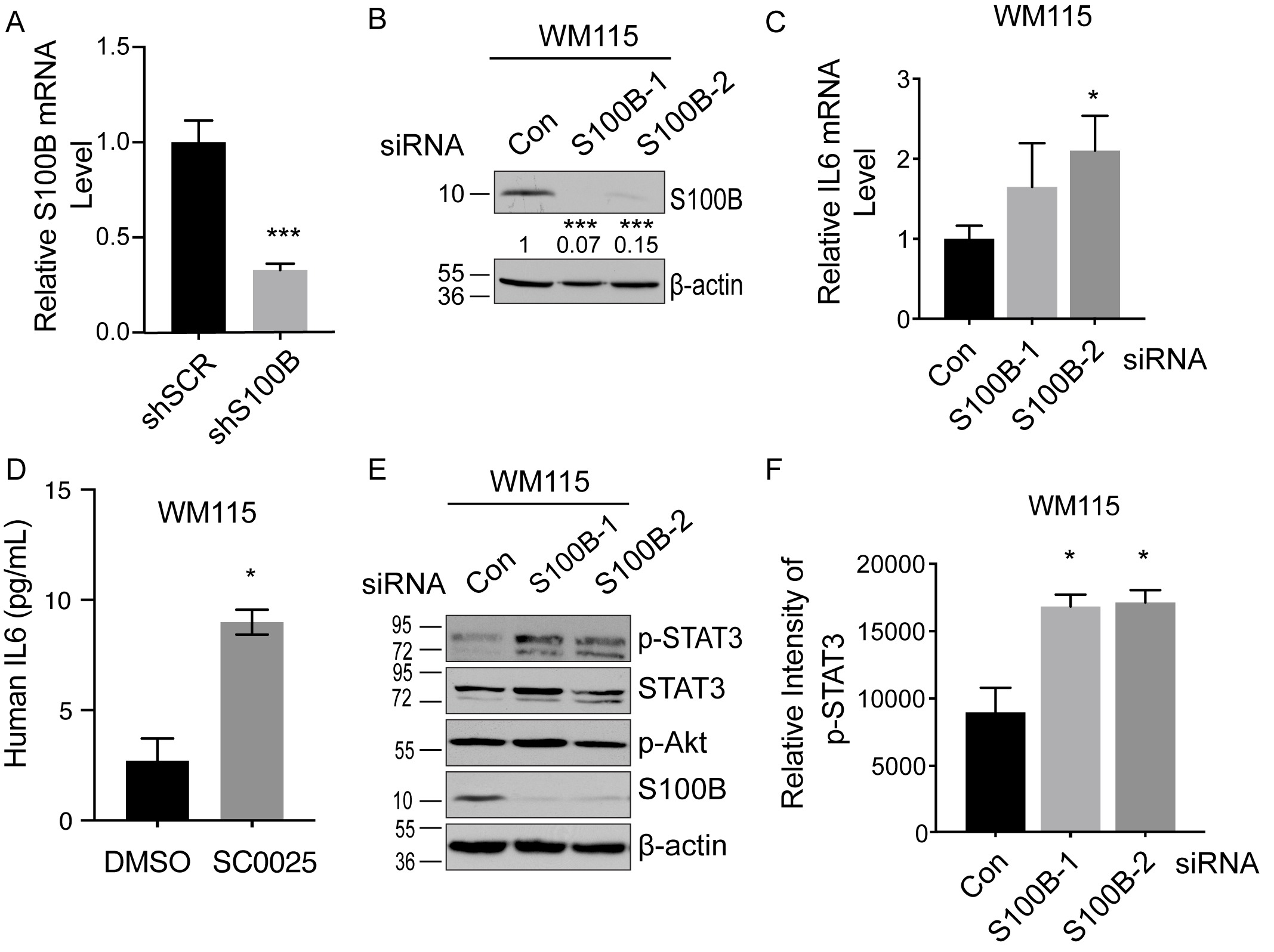

Supplement: S1 Fig — (A) qRT-PCR of the relative S100B mRNA level in shSCR and shS100B WM115 cells (n = 3; mean ± SD; ***, P < 0.005). (B) Immunoblot analysis of S100B in the parental WM115 cells transfected with either control or S100B siRNAs (n = 3; mean ± SD; ***, P < 0.005). Relative S100B protein levels were quantified using Image J. (C) qRT-PCR of the relative IL6 mRNA level in the parental WM115 cells transfected with either control or S100B siRNA #1 and siRNA #2 (n = 3; mean ± SD; *, P < 0.05). (D) IL6 ELISA was used to detect secreted IL6 levels following treatment with either DMSO or S100B inhibitor (SC0025) at 15 μM for 48 hours (n = 3; mean ± SD; *, P < 0.05). (E) Immunoblot analysis of p-STAT3 (Tyr705), total STAT3, p-Akt (Ser473), and S100B in the WM115 transfected with either control or S100B siRNAs. (F) The average values of three independent experiments were plotted using Image J (n = 3; mean ± SD; *, P < 0.05). (TIF) [file pone.0256238.s001.tif]

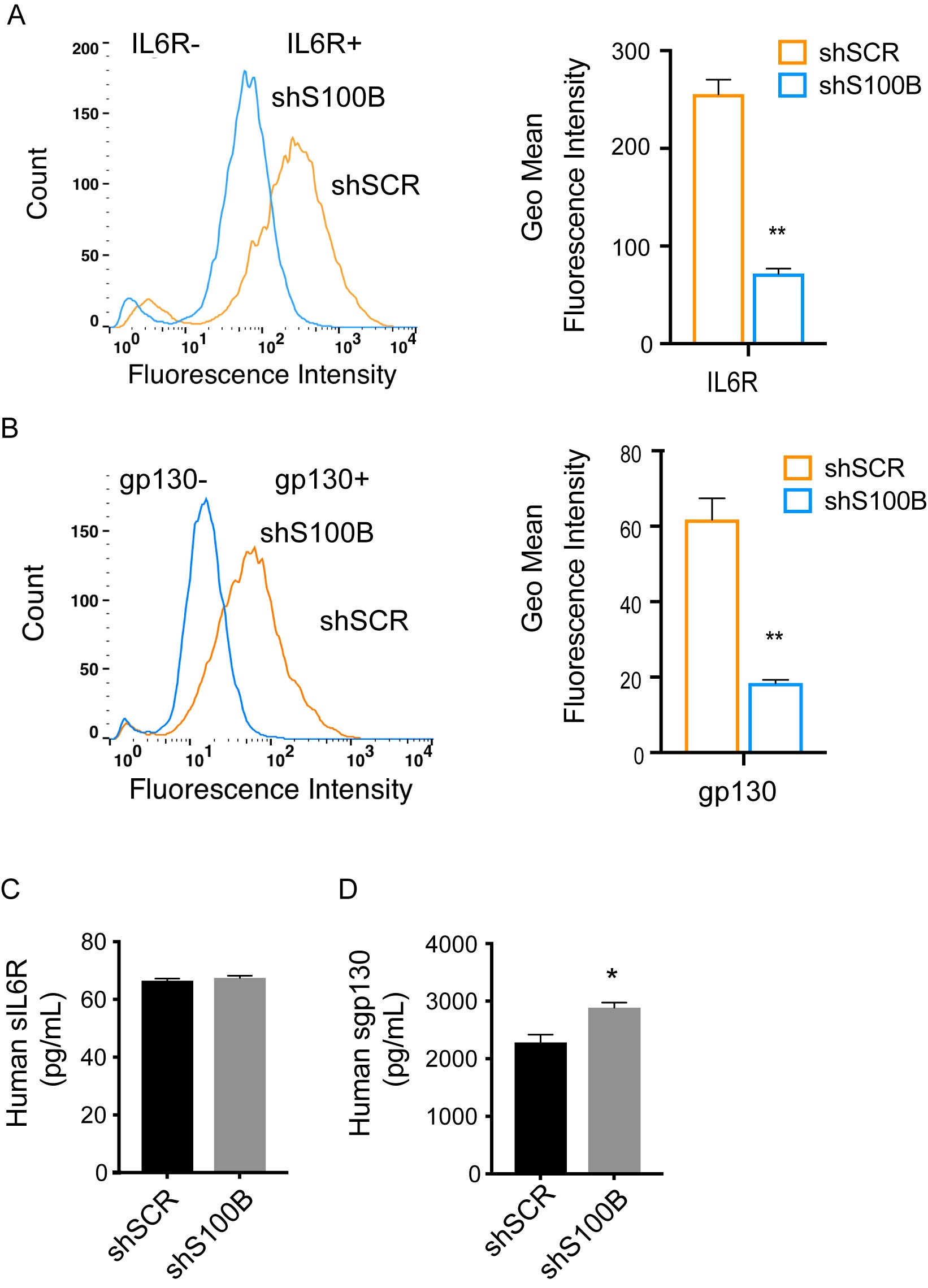

Supplement: S2 Fig — Expression of IL6R (A) and gp130 (B) receptors as determined by flow cytometry in non-targeting scrambled WM115 cells and stable S100B knockdown WM115 cells. Quantitative representation of the flow cytometry analysis (A and B) was done using Prism (n = 3; mean ± SD; ** P <0.005). ELISA was done to determine the secreted sIL6R (C) and sgp130 (D) in the media of the non-targeting scrambled and stable S100B knockdown WM115 cells; n = 2; ± SD; *, P < 0.05). (TIF) [file pone.0256238.s002.tif]

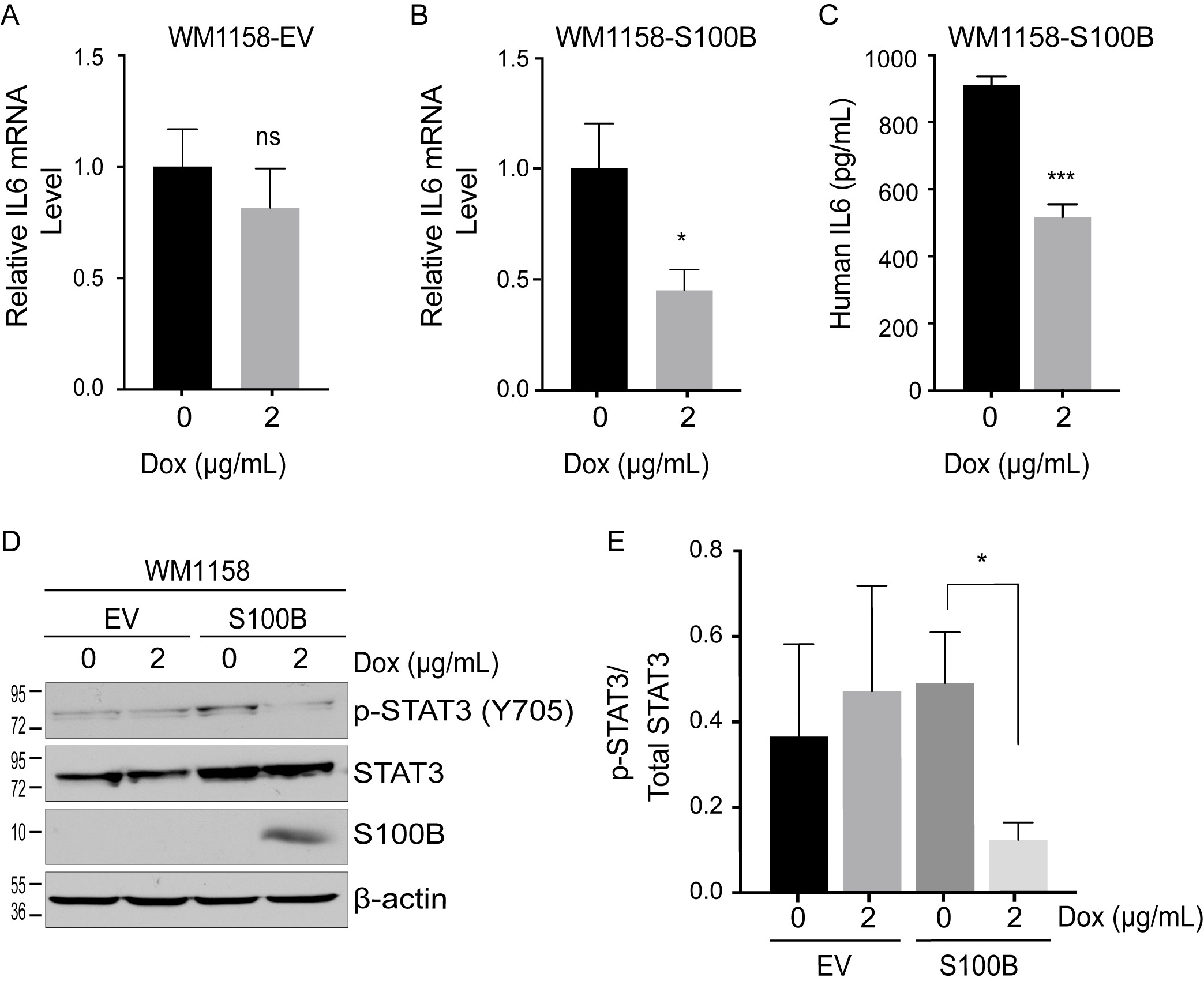

Supplement: S3 Fig — The overexpression of S100B in the WM1158 cells stably expressing a tetracycline-inducible S100B vector (B) shows a significant decrease in relative IL6 mRNA levels via qRT-PCR while an empty vector control (A) had no significant effect (n = 3; mean ± SD.; *, P < 0.05; NS, non-significant). The cells were induced for 72 hours with 2 μg/mL Doxycycline (Dox). (C) ELISA was used to measure secreted IL6 in the WM1158 cells stably expressing tetracycline-inducible S100B vector (WM1158-S100B) showing decreased of secreted IL6 protein level in response to S100B expression (n = 3; mean ± SD.; ***, P < 0.0005). (D) Immunoblot analysis showed an increased S100B upon Dox treatment and decrease in p-STAT3 (Tyr705) with no effect on total STAT3 as shown by the average densitometry measurements of the p-STAT/total STAT3 ratio (E) relative p-STAT3 protein levels were quantified using Image J software (n = 3; mean ± SD; *, P < 0.05). (TIF) [file pone.0256238.s003.tif]

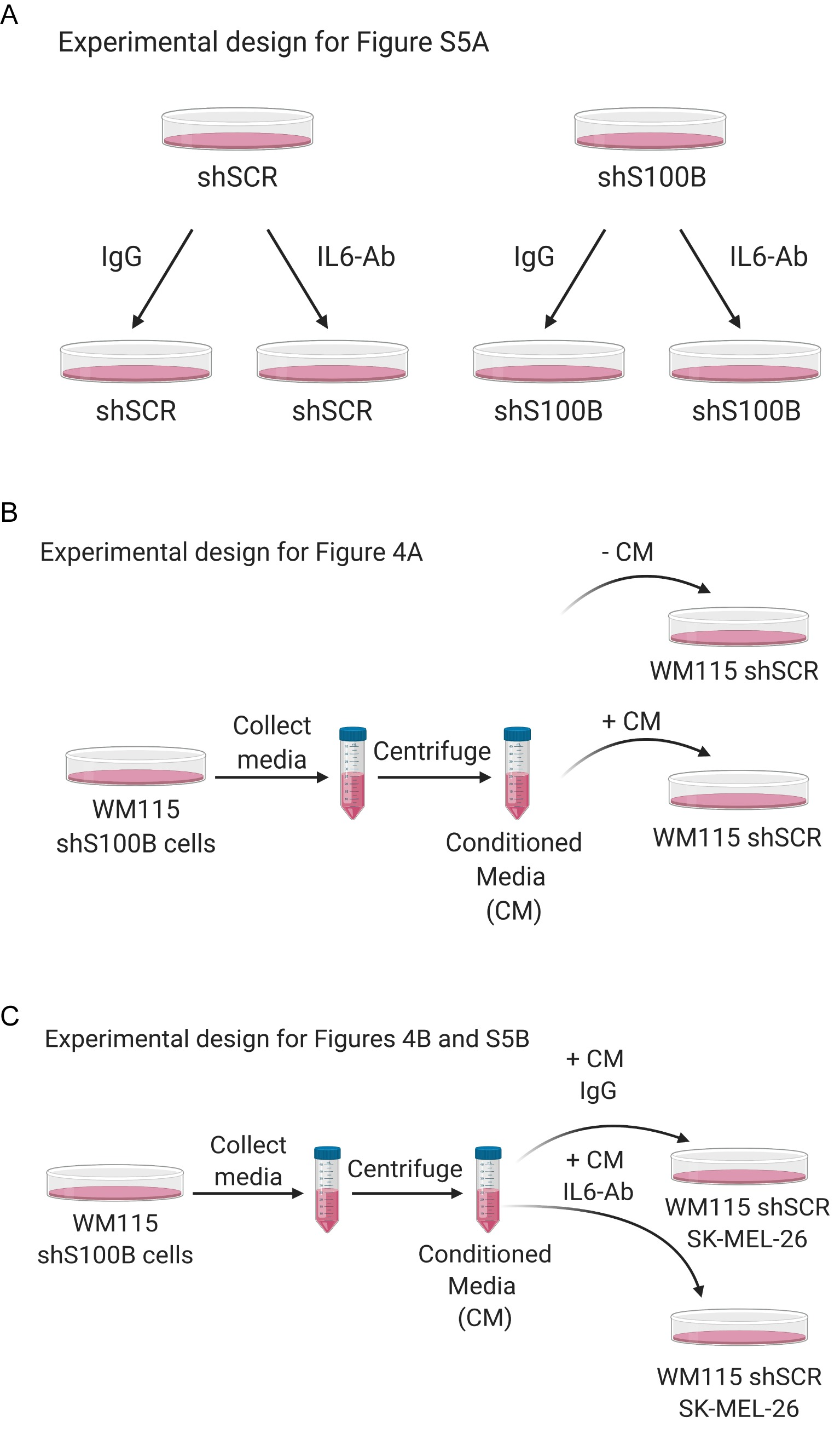

Supplement: S4 Fig — (A) Diagram representing the experimental design for WM115 shSCR and shS100B WM115 cells treated with IgG or IL6-Ab in Fig 4A. (B) Diagram representing the experimental design for the conditioned media (CM) experiment in shSCR WM115 cells in Fig 4B. (C) Diagram representing the experimental design for the conditioned media experiment (CM) in the presence of IgG or IL6-Ab in the shSCR WM115 and SK-MEL-28 cells in Fig 4C and 4D. (TIF) [file pone.0256238.s004.tif]

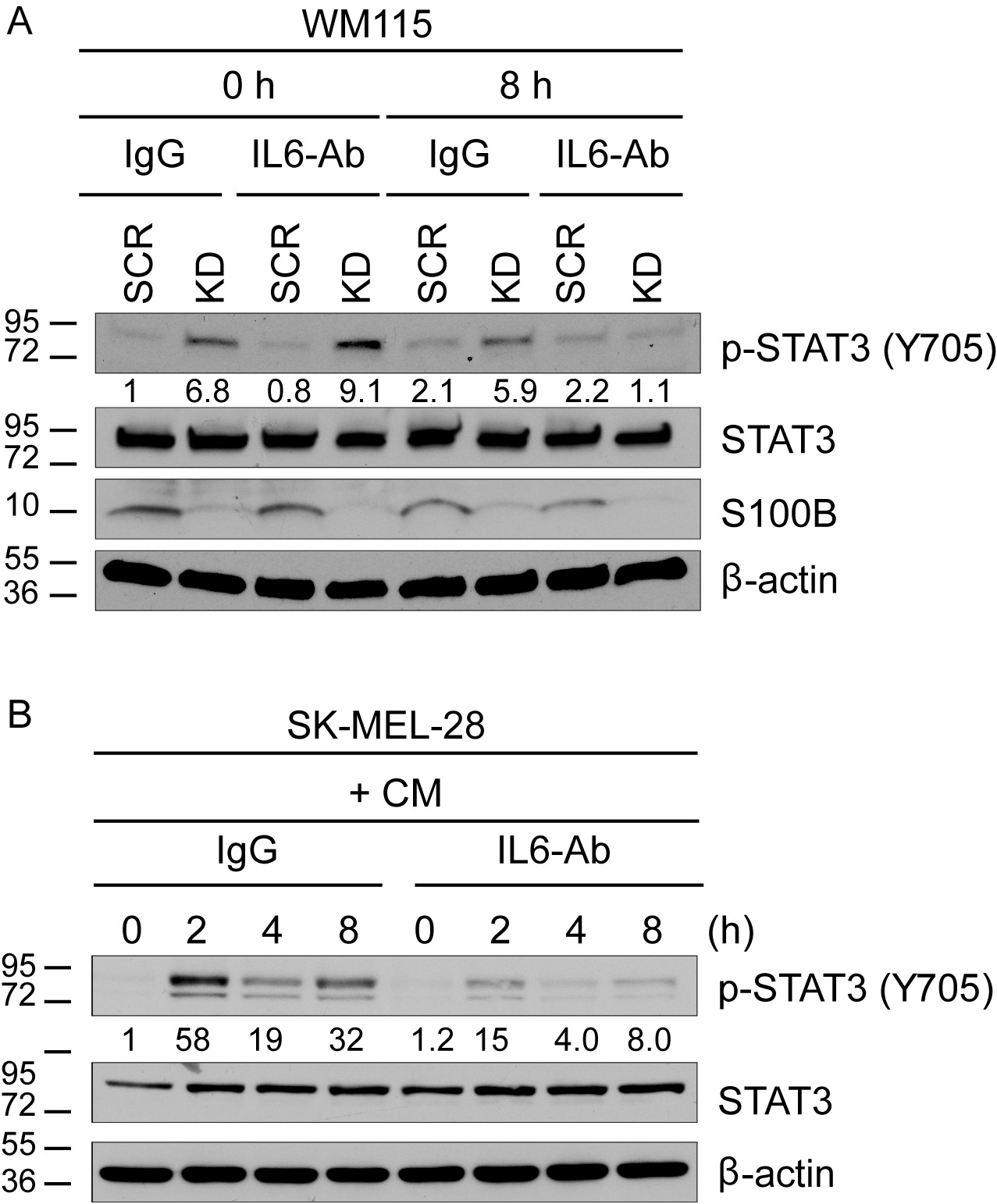

Supplement: S5 Fig — (A) The IL6 inhibitory antibody (0.15 μg/mL) prevented the autocrine activation of p-STAT3 upon addition to shS100B WM115 cells growth media compared to shSCR WM115. After 8 hours, the IL6 Ab treated shS100B cells did not have the increased p-STAT3 seen in the control IgG treated shS100B cells. (B) SK-MEL-28 cells were cultured with the shS100B WM115 CM in the absence/presence of an IL6 Ab (0.15 μg/mL). Immunoblot analysis of p-STAT3 (Tyr705) and total STAT3 after 0, 2, 4, and 8 hours of treatment shows the IL6 Ab prevents increased p-STAT3 production. Relative p-STAT3 protein levels were quantified using Image J software. Blot is a representative of two independent replicates. (TIF) [file pone.0256238.s005.tif]

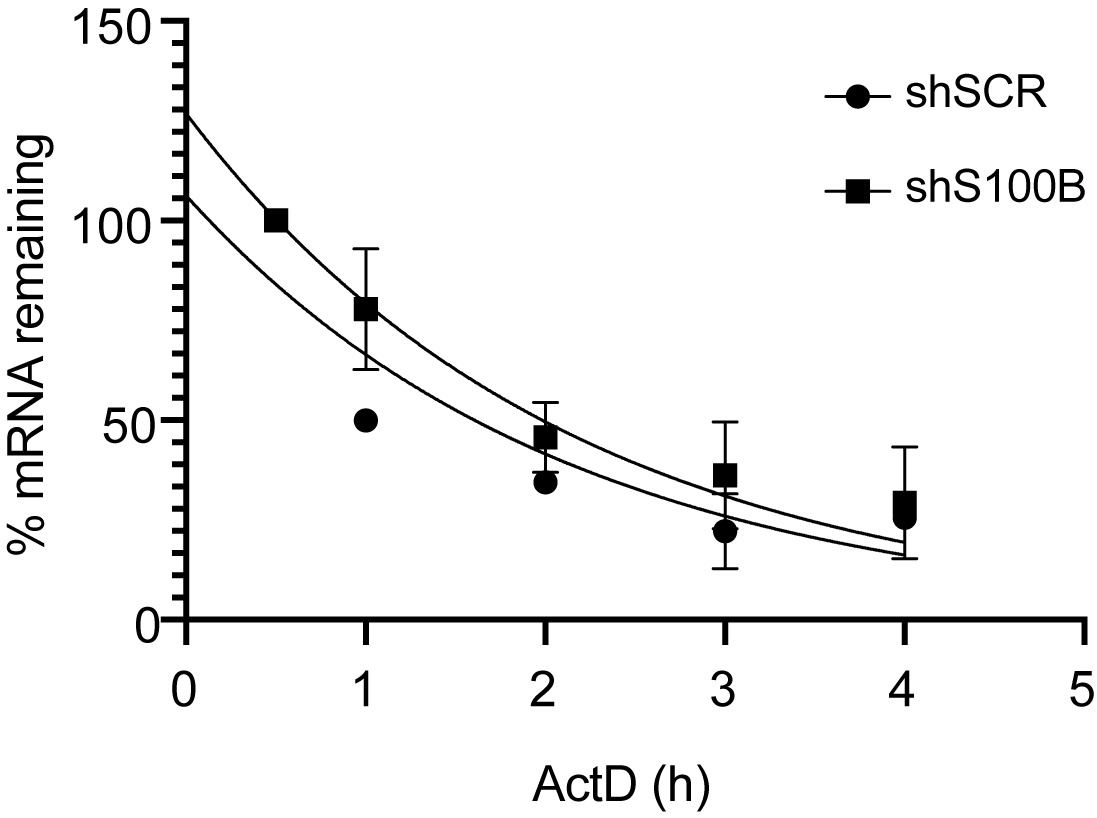

Supplement: S6 Fig — The mRNA half-life of IL6 mRNA was measured in the non-targeting scrambled (shSCR) and S100B stable knockdown (shS100B) cell lines by Actinomycin D assay. The percentage of IL6 mRNA remaining was plotted as a function of time following transcription inhibition by Act D. Plot is an average of two independent biological replicates. The data were fit to one phase exponential decay using Prism 8.0 to calculate the half-life of IL6 mRNA. shSCR half-life: 1.2 h ± 0.4 h; shS100B half-life: 1.7 h ± 0.3 h; P = 0.1908. The standard errors were calculated from the 95% confidence intervals. The goodness of fit with R2 for the rates was 0.84 (shSCR) and 0.88 (shS100B). (TIF) [file pone.0256238.s006.tif]

Fig 1C

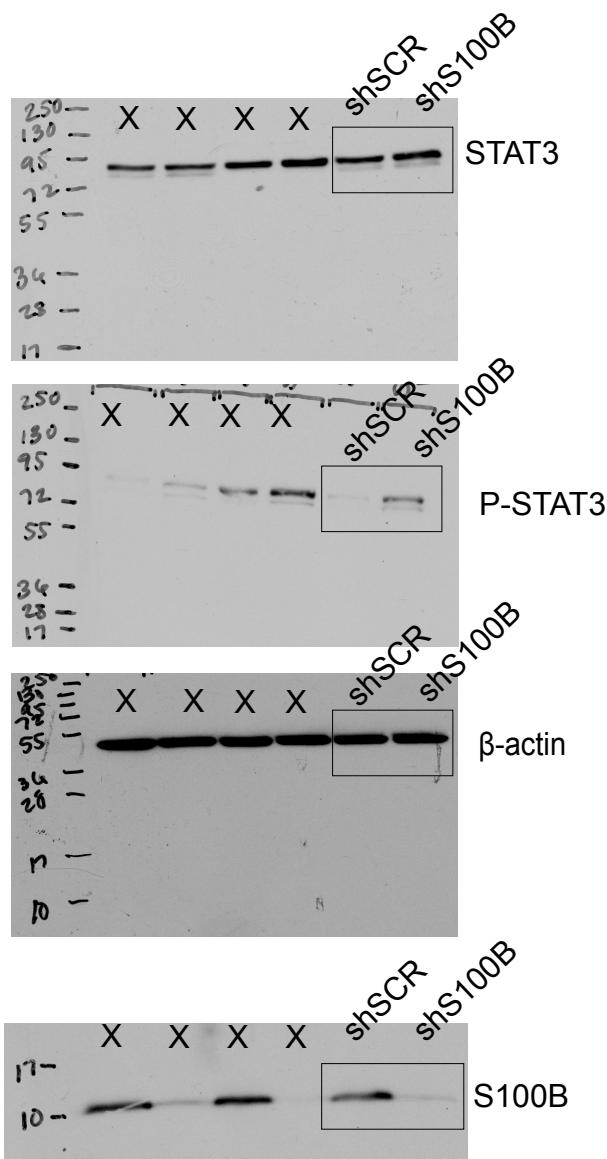

Fig 2C

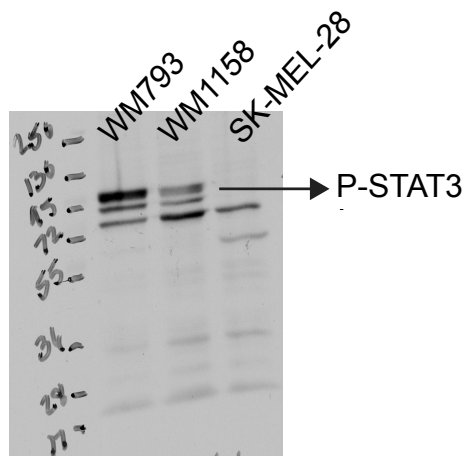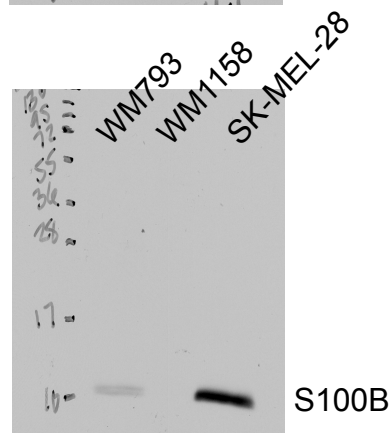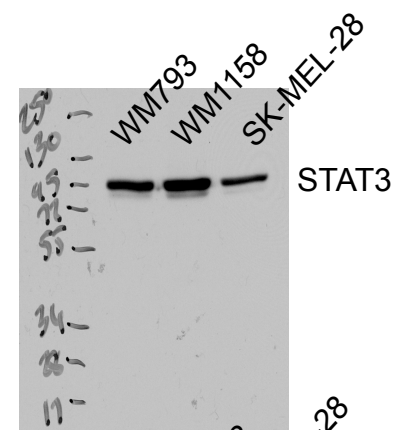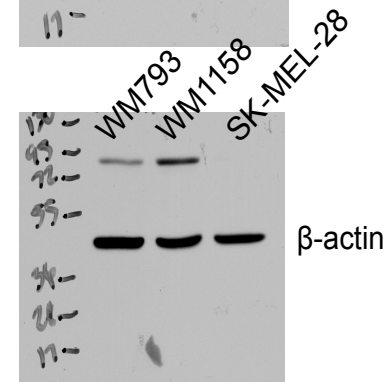

Fig 2D

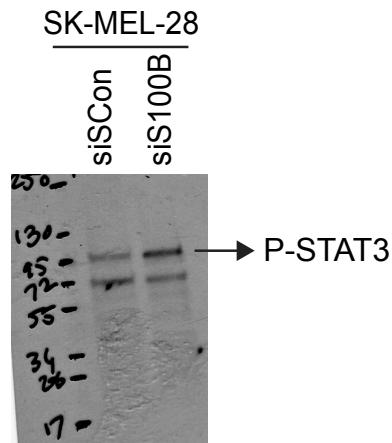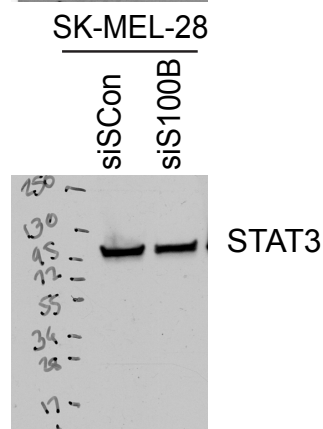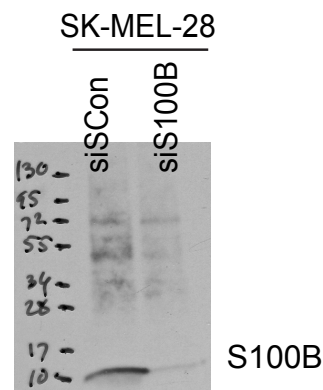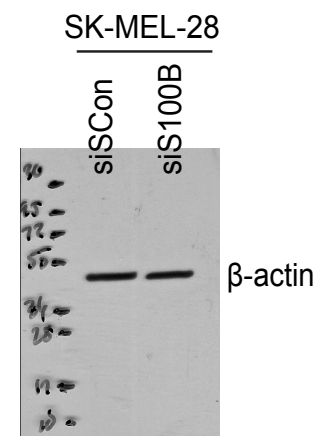

Fig 3D

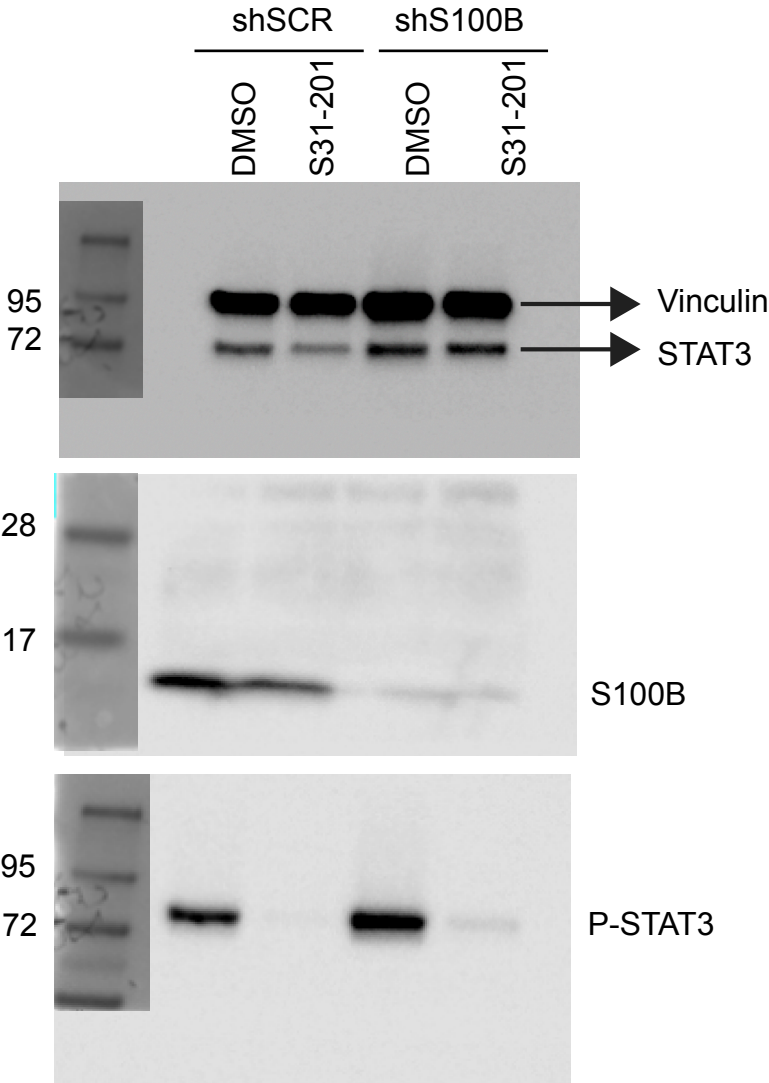

Fig 4A

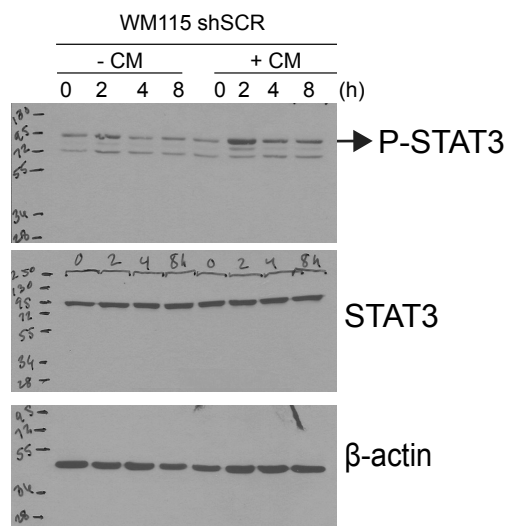

Fig 4B

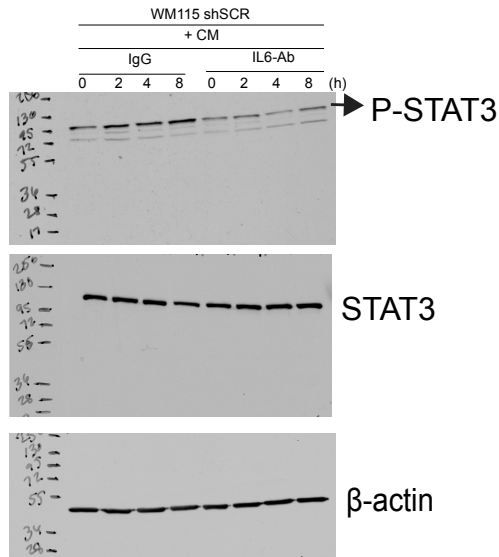

Fig 5C

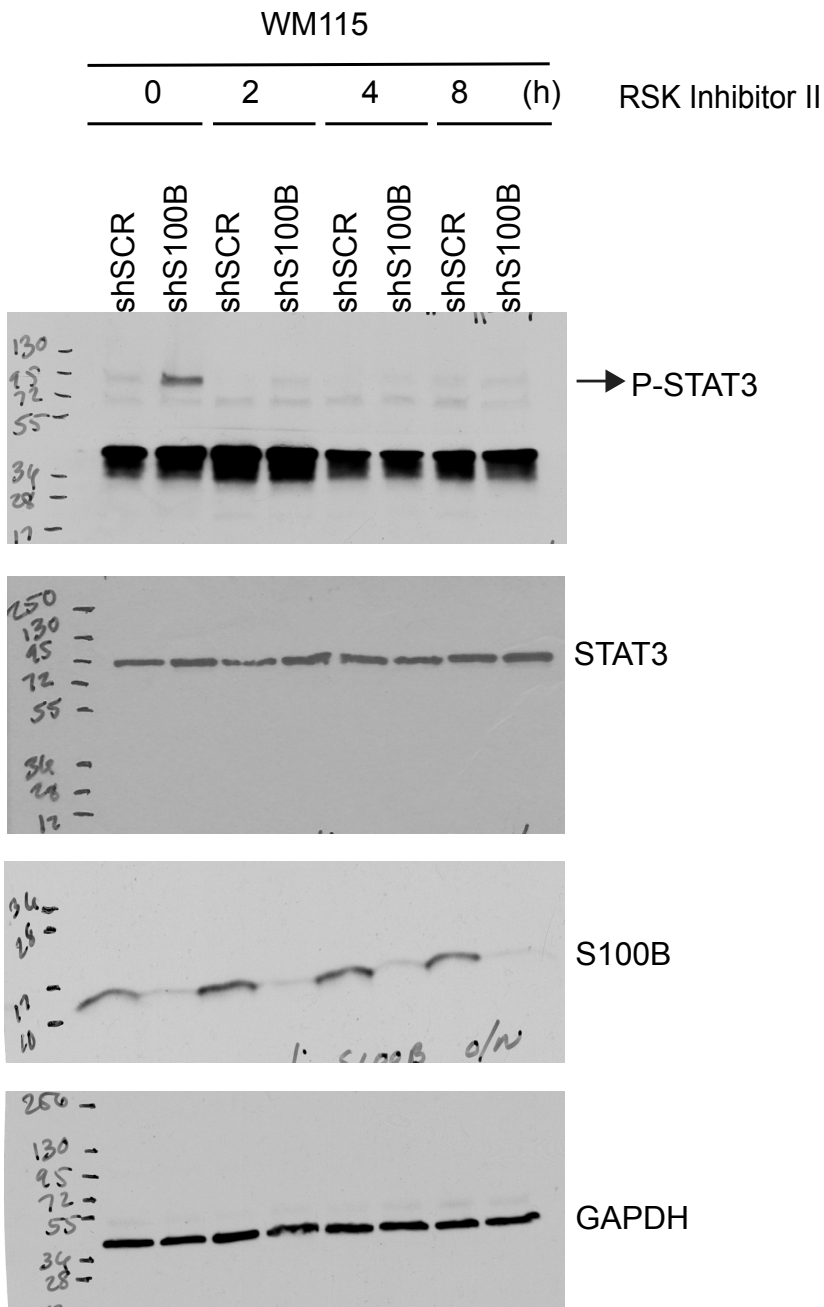

Fig 6A

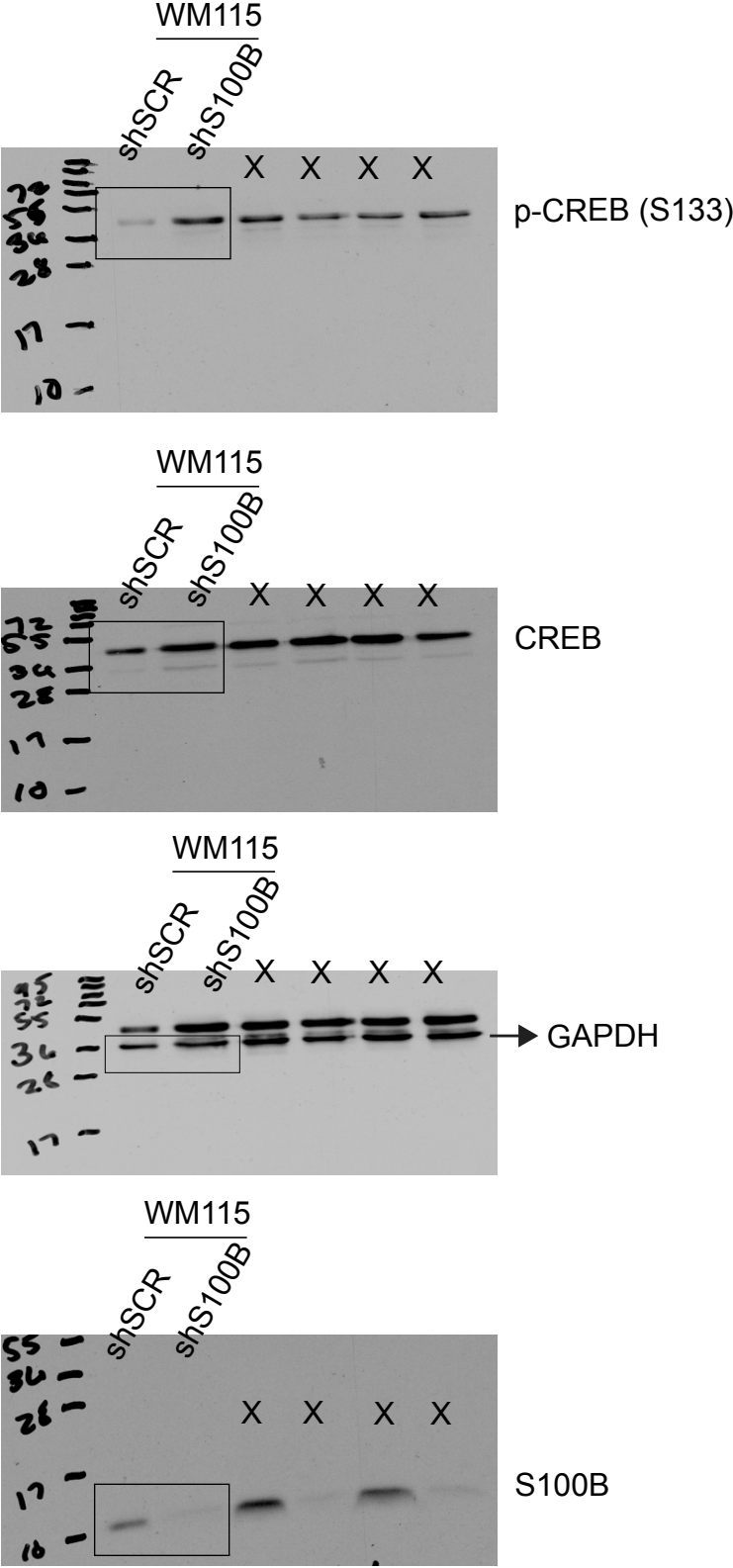

Fig 6D

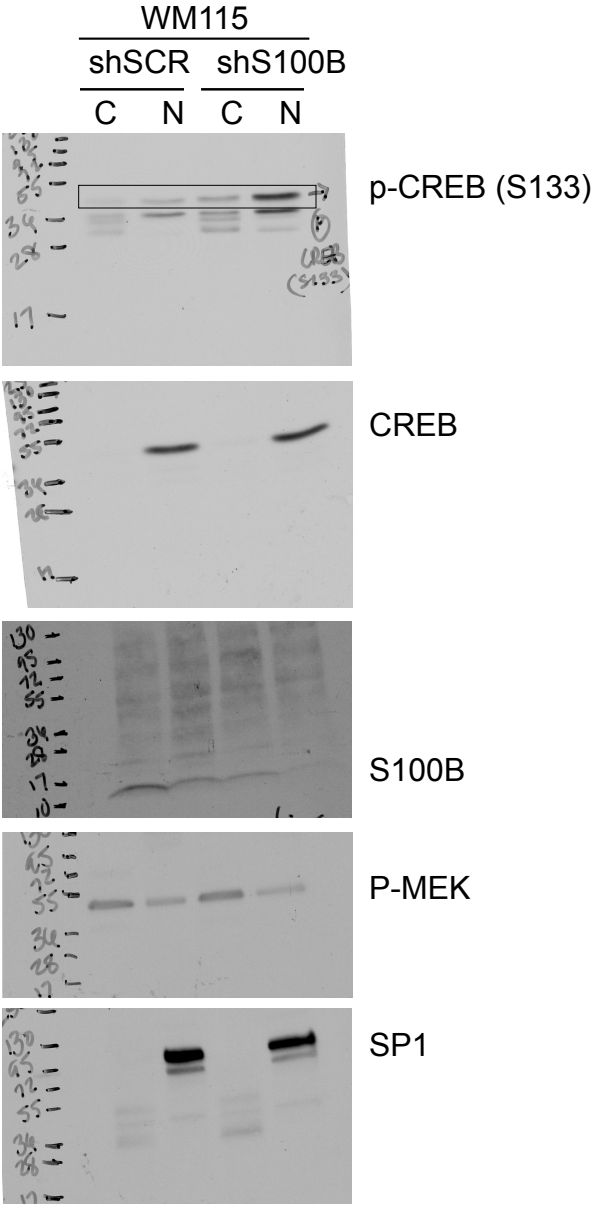

Fig 6E

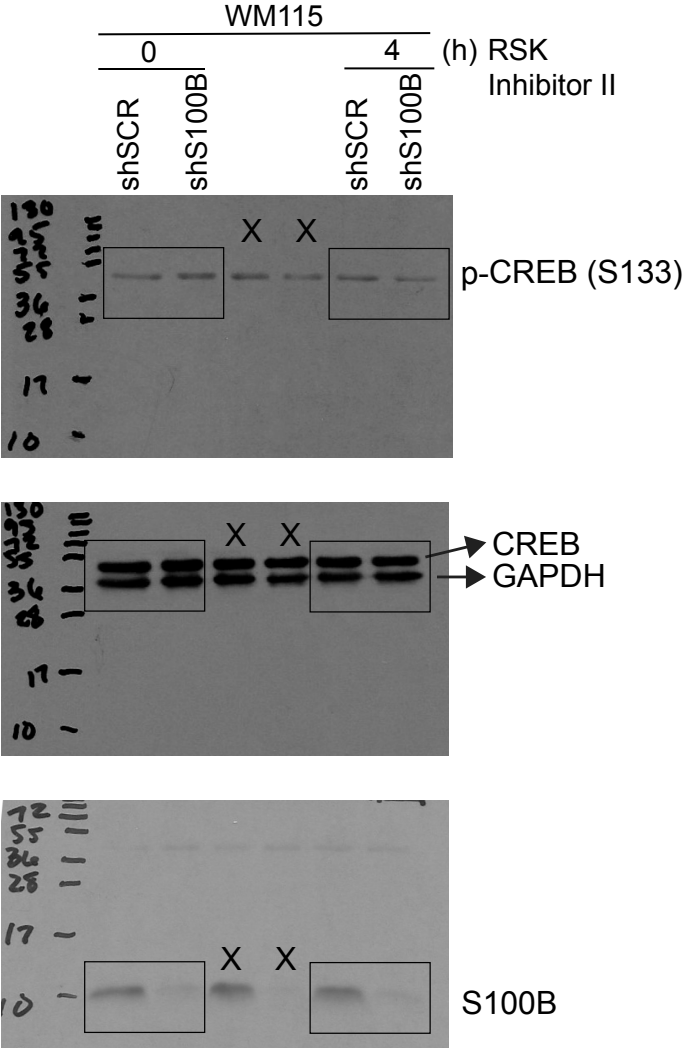

Fig 6G

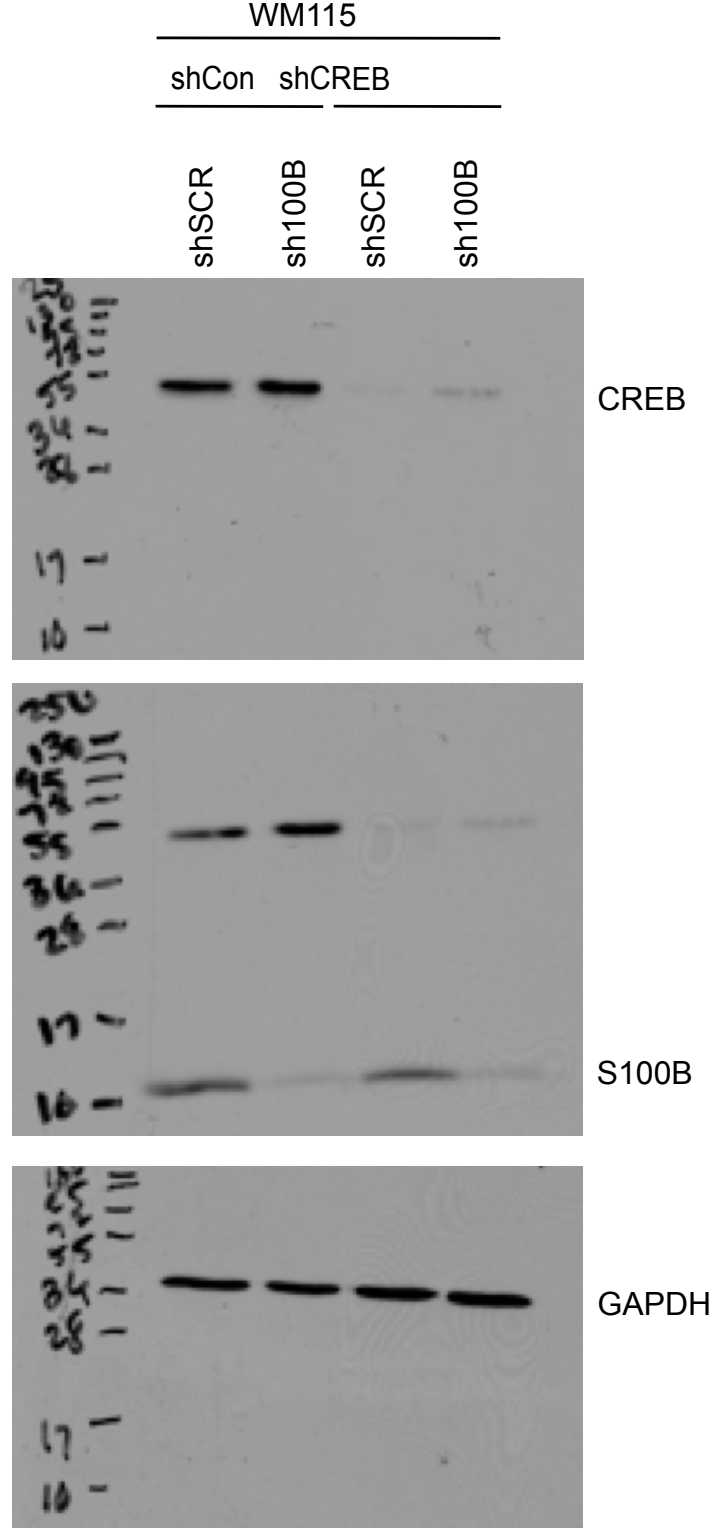

S1B Fig

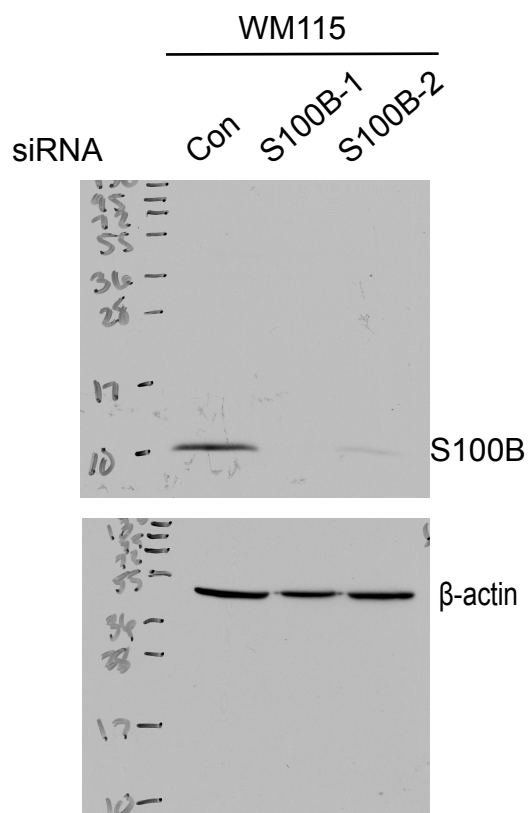

S1E Fig

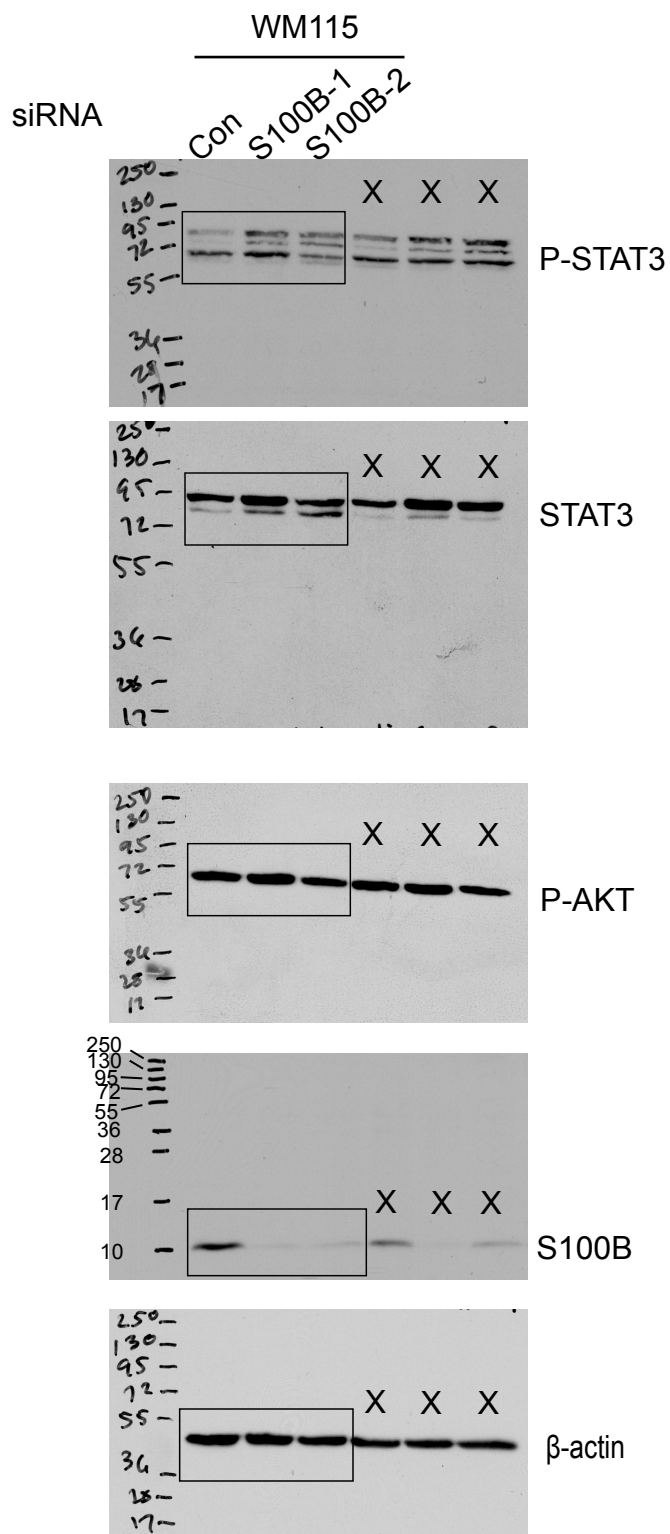

S3D Fig

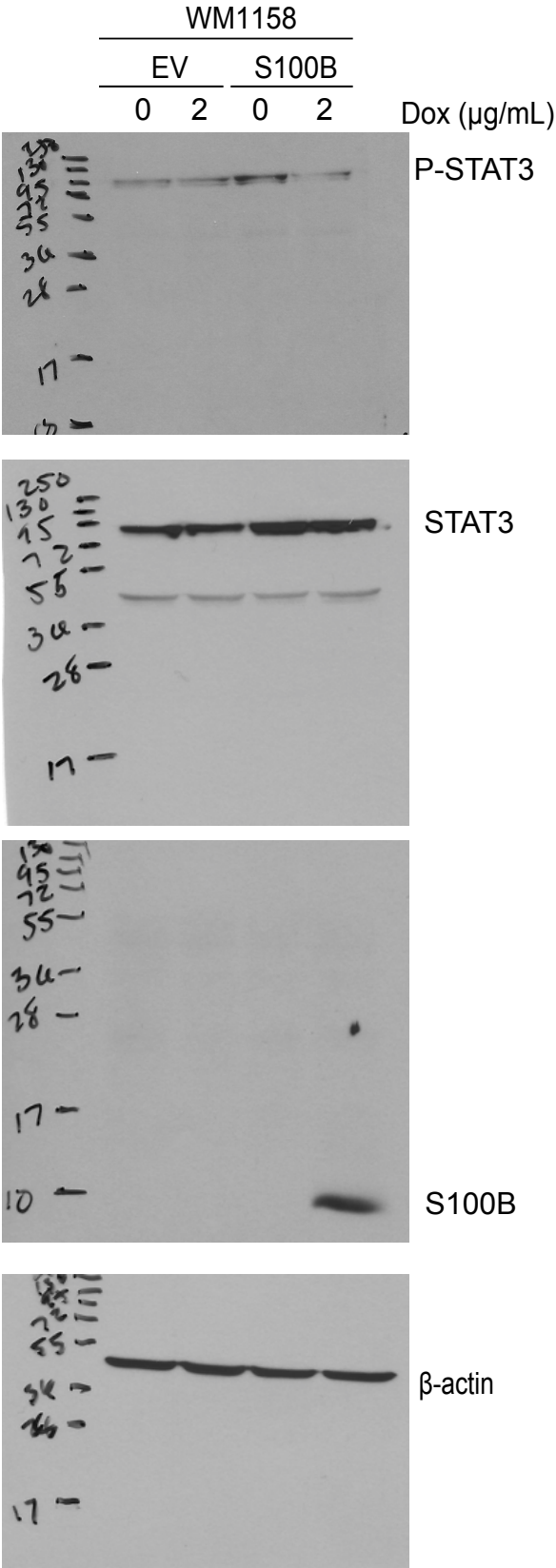

S5A Fig

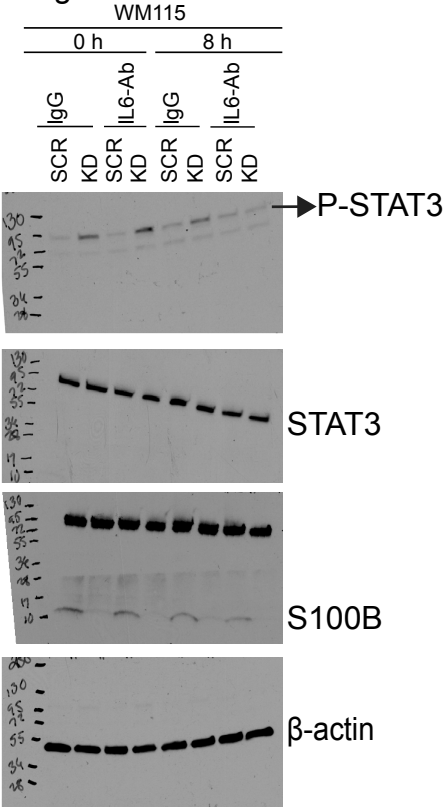

S5B Fig

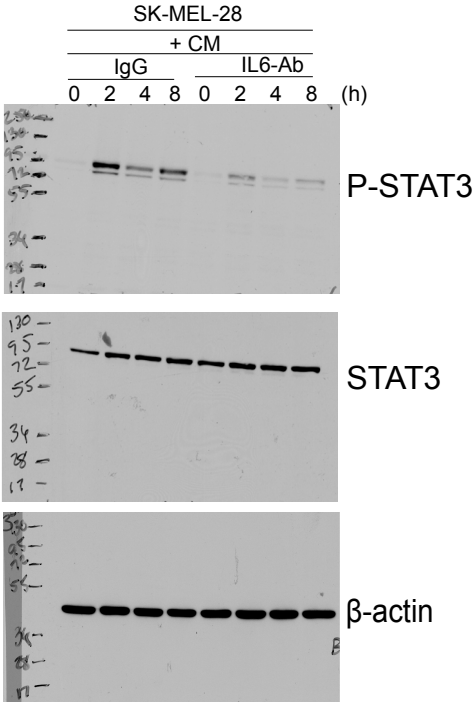

Supplement: S1 Raw Images — (PDF) [file pone.0256238.s007.pdf]
